# Designing Amino Functionalized Titanium-Organic Framework on Separators Toward Sieving and Redistribution of Polysulfides in Lithium-Sulfur Batteries

**DOI:** 10.1007/s40820-025-01733-0

**Published:** 2025-05-26

**Authors:** Xiaoya Kang, Tianqi He, Hao Dang, Xiangye Li, Yumeng Wang, Fuliang Zhu, Fen Ran

**Affiliations:** State Key Laboratory of Advanced Processing and Recycling of Non-Ferrous Metals, School of Materials Science and Engineering, Lanzhou University of Technology, Lanzhou, 730050 People’s Republic of China

**Keywords:** Size–sieving effect, Functional separator, MOF, Polysulfides shuttle, Lithium–sulfur batteries

## Abstract

Shuttle effect of polysulfides overshadows the superiorities of lithium–sulfur batteries. Size–sieving effect could address this thorny trouble rely on size differ in polysulfides and lithium ions. However, clogged polysulfides pose some challenges for cathode and are rarely recycled during charging/discharging. Herein, an amino functionalized titanium-organic framework is designed for modifying lithium–sulfur batteries separator to address the aforementioned challenges. Wherein, the introduction of amino narrows titanium–organic framework pore size, enabling functional separator to selectively modulate lithium ions and polysulfides migration using size-sieving effect, thereby completely suppressing polysulfides shuttle. Furthermore, the blocked polysulfides will be adsorbed on the separator surface by positively charged amino leveraging electrostatic adsorption, ensuring polysulfides to redistribute and reuse, and boosting active materials utilization. Significantly, the migration of lithium ions is not hindered since there are lithium ions transfer channels formed via Lewis acid–base interaction with the help of amino. Combined with these virtues, the lithium–sulfur batteries with amino functionalized titanium-organic framework modified separator enjoy an ultralow attenuation rate of 0.045% per cycle over 1000 cycles at 1.0C. Electrostatic adsorption and Lewis acid–base interaction cover deficiencies existing in the inhibition of polysulfides shuttle by size-sieving effect, providing fresh insight into the advancement of lithium-sulfur batteries.

**Supplementary Information:**

The online version contains supplementary material available at 10.1007/s40820-025-01733-0.

## Highlights

- Amino could narrow the sub-nanopore size of Ti–MOF modified layer to around 0.8 nm for perfectly inhibiting polysulfides shuttle.
- Amino enables redistribution and reutilization of blocked polysulfides via leveraging electrostatic adsorption.
- Electrostatic adsorption and Lewis acid–base interaction make up for the deficiency of size effect in inhibiting polysulfides shuttle.

**Supplementary Information:**

The online version contains supplementary material available at 10.1007/s40820-025-01733-0.

## Graphical abstract

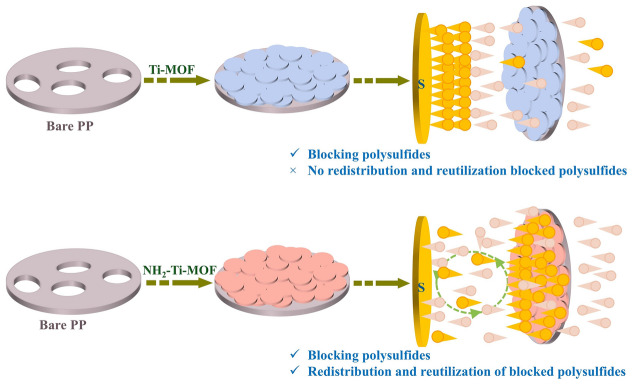

## Introduction

The energy crisis is driving us to look for novel, clean energy storage devices [1–3]. Rechargeable lithium–sulfur (Li–S) batteries have held considerable promise as next–generation high–energy–storage batteries owing to the superiorities of sulfur cathode [4, 5]. Cooperated with Li, sulfur can deliver ultra–high theoretical energy densities (2800 Wh kg^−1^) and superior theoretical capacity (1675 mAh g^−1^) [6, 7]. Regrettably, the development of Li–S batteries is plagued by a series of issues caused the “shuttle effect” of polysulfides, such as continuous depletion of sulfur and self–discharging. [8, 9]. Also, polysulfides that traverse the macropores of polypropylene (PP) separator would adhere to lithium metal surface, resulting in corrosion and passivation [10, 11]. This ultimately leaves rapid capacity fading and a shorter cycle lifespan of Li–S batteries [12, 13]. Therefore, it is imperative to suppress polysulfides migration for perfectly delivering the merits of Li–S batteries.

Although commercial separator that features wealthy pore architecture ensures Li^+^ migration during charging/discharging, the larger pore size is impossible to block the shuttle of polysulfides [14, 15]. Considering the difference in size of solvated polysulfides and Li^+^, exploiting functionalized coating to regulate selectively ion migrations in separators, that is, employing size–sieving effect, is a straightforward and effective approach to inhibit the shuttling of polysulfides. Delightfully, the size-sieving effect is highly effective in inhibiting polysulfides shuttle without hampering Li^+^ transport. Whereas, it should be stressed that the ion–selective channel of separator modified layer is supposed to be around 0.8 nm, which has been verified in our previous work [16, 17]. Therefore, selecting the appropriate materials to modify separator is a critical step.

Metal–organic framework (MOF), as uniquely porous materials characterized by well–defined and adjustable pore sizes and tunable surface chemistry, enable selectively separate various ions and molecules [18, 19]. Such as 2D zeolitic imidazolate framework films are used for highly permeable selective H_2_ separation [20], and functionalized UiO–66–(X)_2_ membrane could high–selectively separate monovalent and divalent cations [21]. Additionally, a novel MOF–gel film, as a permselective separator, is employed to restrain adverse reactions of soluble intermediates, which achieves a much longer cycle life of rechargeable organic batteries [22]. Undeniably, this brilliant selective separation ability of MOF also exerts an unsurpassed role in controlling the selective migration of ions in Li–S batteries. The affluent sub-nanometer pore size inhibits polysulfides shuttle firstly using size–sieving effect if MOF is employed as separator modified layer, and even pore structure is also favorable for uniform and fast Li^+^ transport. Simultaneously, the high polarity of MOF gives them a relatively high affinity for electrolyte, complementing the inferior wettability of the Celgard separator. For instance, UiO–66 with narrow pore size distribution of 0.5–0.6 nm is employed as ion sieving coating to inhibit polysulfides shuttle, thereby realizing pleasurable electrochemical behaviors with a low–capacity decay rate of 0.08% after 200 cycles [23]. Another, the mixed matrix membrane prepared with UiO–66–SO_3_Li and poly(vinylidene fluoride) is introduced into separator for Li–S batteries to inhibit polysulfides migration by virtue of size effect and electrostatic repulsion [24]. Similarly, an anionic MIL–101–SO_3_H based separator is engineered to restrain the migration of polysulfides toward anode [25]. Recently, Zr–MOF and In/Zr–MOF with pore sizes of 1.41 and 1.20 nm, respectively, are employed for separator modification. Combining the size effect and catalytic effect, the performances of Li–S batteries assembled with Zr–MOF and In/Zr–MOF modified separators are greatly enhanced [26, 27]. However, it is noted that these works simply utilized the sieving effect of MOF to hinder the migration of polysulfides toward anode without considering the redistribution and reuse of blocked polysulfides at cathode side. It is the fact that polysulfides, which are suppressed by size effect, would cover the surface of cathode, resulting in the blockage of host materials’ pores, thus affecting the electrical conductivity of electrode materials as well as the wettability of the electrolyte. In addition, the accumulation of polysulfides also prevents Li^+^ from contacting the cathode active material, thereby impairing the further discharging of Li–S batteries. Thus, it is crucial to further refine size effect strategy to redistribute the blocked polysulfides so that they are capable of being reused during charging/discharging. Meanwhile, the neglected aspect of Li^+^ migration in MOF pores should also be given due attention.

In view of this, the NH_2_–Ti–MOF is meticulously designed and prepared for separator coating of Li–S batteries so as to deliver a solution for the aforementioned challenges. The polar functional group –NH_2_ in MOF exerts three contributions in inhibiting polysulfides shuttling: i) Size–sieving effect. The –NH_2_ group narrows the sub-nanopore size of Ti–MOF from 0.95–1.10 to 0.83–0.86 nm, which can perfectly inhibit polysulfides shuttle while not hindering Li^+^ migration. ii) Electrostatic adsorption. The blocked negatively charged polysulfides could be adsorbed by –NH_2_ group with a positively charged, ensuring a more exhaustive discharging of cathode active material. Moreover, polysulfides adsorbed on the separator surface can be returned to cathode and electrochemically reduced during charging, thus improving the sulfur availability. iii) Lewis acid–base interaction. The acid–base interactions between Li^+^ and the –NH_2_ group can facilitate the formation of directed and fast Li^+^ transfer channels. Based on above merits, the Li–S batteries assembled with NH_2_–Ti–MOF modified separator deliver a favorable capacity of 1, 079.8 mAh g^−1^ at 0.5C, an ultralow attenuation rate of 0.045% per cycle over 1000 cycles at 1.0C. Electrostatic adsorption and Lewis acid–base interaction make up for the deficiency of size effect in inhibition polysulfides shuttle, providing fresh insight into the advancement of Li–S batteries.

## Experimental Section

### Materials

Terephthalic Acid (H_2_BDC) was produced by Tianjin Energy Co., Ltd. (China). 2–aminoterephthalic acid (2–NH_2_–H_2_BDC) and titanium isopropoxide (TPOT) were supplied by Shanghai Macklin Biochemical Technology Co., Ltd. (China). N, N–dimethylformamide (DMF) was purchased from Tianjin Damao Chemical Reagent Co., Ltd. (China). Methanol was given by Tianjin Industrial Company (China). N–methylpyrrolidone (NMP) was obtained from Aladdin Reagent Co., Ltd. (China). Ketjenblack, super P (Li), and sublimed sulfur were provided by Guangdong Canrd New Energy Technology Co., Ltd. (China). The Li–S electrolyte with 1 M lithium bis (trifluoromethanesulfonyl)—imide (LiTFSI) in a mixed solvent of 1, 3–dioxolane (DOL) and 1,2–dimethoxyethane (DME) addition of 2 wt% LiNO_3_, 0.1 M Li_2_S_6_ in DME: DOL = 1: 1 vol%, and the solvent of DME: DOL = 1: 1 vol% were acquired by Suzhou DoDo Chemical Technology Co., Ltd. (China). Hexadecane was bought from Shanghai Zhanyun Chemical Co., LTD. Deionized water was self–provided by the laboratory. All chemical reagents were used without any additional treatment.

### Preparation of NH_2_–Ti–MOF and Ti–MOF Materials

The NH_2_–Ti–MOF–0 material was designed and prepared as follows: 1.98 g H_2_BDC was dissolved in 67.5 mL of DMF and stirred until thorough dissolution. Meanwhile, 1.62 mL TPOT slowly dropped in 7.5 mL methanol under vigorous stirring to form a milky white solution. Subsequently, the latter containing titanate solution was added drop by drop to the former containing organic linker solution under vigorous stirring for 2 h. Afterward, the mixture solution was transferred to a Teflonlined stainless steel autoclave and heated at 150 °C for 24 h. After naturally cooling to room temperature, the resulting yellow precipitates were collected via centrifugation and then washed with DMF and methanol in turn. Finally, the product was dried in an oven at 80 °C for 12 h and denoted as NH_2_–Ti–MOF–0. Ti–MOF–0 was synthesized as similar routing. The NH_2_–Ti–MOF and Ti–MOF materials were gained by heating treatment of as-obtained NH_2_–Ti–MOF–0 and Ti–MOF–0 at 250 °C for 2 h under N_2_ atmosphere.

### Preparation of MOF–Modified Separators

Initially, the PVDF was fully dissolved in NMP, followed by adding the mixture MOF powder and super P with a weight ratio of 7: 2: 1 (MOF: super P: PVDF) and stirring gently for 14 h. Then, the slurry was evenly coated on the one side of commercial PP separator (Celgard 2500) and vacuum–dried at 55 °C for 14 h. Eventually, the as–obtained MOF/PP membrane was cropped into the 19 nm diameter disks with MOF approximately 0.37–0.50 mg cm^−2^.

### Preparation of Ketjenblack/S Composite and Ketjenblack/S Cathode

The Ketjenblack/S composite was prepared via the conventional melt–diffusion approach. Concretely, the Ketjenblack was mixed and ground together with sublimed sulfur (S) in a mass ratio of 1: 3 and then the mixture was heat-treated at 155 °C for 12 h under Ar atmospheric condition. Subsequently, 80 wt% Ketjenblack/S, 10 wt% super P, and 10 wt% PVDF binder were blended in a small amount of NMP solvent and stirred for 14 h to generate a homogeneous slurry, and it was homogeneously cast on a one-sided carbon-coated aluminum foil and vacuum dried at 55 °C for 14 h to get Ketjenblack/S cathode. The mass loading of sulfur was about 0.84–1.0 mg cm^−2^.

### Electrochemical Performance Measurements

The electrochemical measurements were performed using the CR2032 coin–type batteries with Ketjenblack/S served as cathode, lithium metal (the thickness is about 0.45 mm) served as anode, and modified separators served as separator. The 1.0 M LiTFSI dissolved in the mixture solvent of DOL/DEM (*V*: *V* = 1: 1) with 1.0 M LiNO_3_ was employed as electrolyte. Cycling performances, and galvanostatically charging/discharging (GCD) of coin-type batteries were all performed on LAND CT2001A system within 1.7–2.8 V potential intervals. The cyclic voltammetry (CV) and electrochemical impedance spectra (EIS) curves were recorded employing CHI660E electrochemical workstation.

### Measurement of Ionic Conductivity

The symmetric batteries were constructed via the clamping of diverse separators between two same–sized stainless–steel electrodes. The EIS measurements were conducted on an open circuit of 5 mV potential in a frequency interval of 0.1–10^6^ Hz. The ionic conductivity (*σ*, mS cm^−1^) of MOF–modified separators or PP separator were evaluated according to Eq. (1):

1 $$\sigma = L/(R \times A)$$

where *L* mean the thickness of separators (cm), *R* signified the resistance (Ω), and *A* was on behalf of the area of the stainless–steel electrode (cm^2^).

### Measurement of Li^+^ Transference Number

Symmetrical batteries were assembled by sandwiching the NH_2_–Ti–MOF, and Ti–MOF coated separators or PP separators, respectively, between two Li–Li electrodes. Testing AC impedance and DC potentiostatic polarization measurements of symmetrical batteries by using electrochemical working station (CHI660E) to gain steady state current to initial state current. The Li^+^ transference numbers of MOF–modified separators were defined as the ratio of the two by Eq. (2):

2 $$t_{{(Li^{ + } )}} = I_{s} /I_{0}$$

where *t*_(Li+)_ stood for the transference number of Li^+^, and *I*_0_ and *I*_s_ denoted the initial and stable state current, respectively.

### Measurements of Dynamic Contact Angle, Electrolyte Uptake, and Electrolyte Retention

The dynamic contact angle of MOF–modified separators or PP separator were conducted on the surface of separators utilizing lithium–sulfur batteries electrolyte at room condition.

The initial mass of MOF–modified separators or PP separator was labeled as *m*_*1*_, subsequently it was immersed in electrolyte and taken out at certain time intervals to be weighed and labeled as *m*_*t*_. The electrolyte uptake was evaluated according to Eq. (3):

3 $$Electrolyte \,\,uptake (\% ) = (m_{t} - m_{1} )/m_{1} \times 100 \%$$

The MOF-modified separators or PP separator was immersed in the electrolyte for 2 h followed by taking out and weighing mass denoted as m_1_, then left to rest for a certain period of time at room temperature and weighed its mass denoted as m_t_. Electrolyte retention can be assessed by Eq. (4):

4 $$Electrolyte\,\,retention\;\; (\% ) = (m_{t} - m_{1} )/m_{1} \times 100 \%$$

### Measurement of Porosity

The well–cut separators were soaked in hexadecane for 2 h and then taken out, accompanied by wiping off the unabsorbed electrolyte on surfaces using filter paper. The porosity of separators was evaluated by the below equation with the help of masses of separators before and after immersion.

5 $$Porosity\;\; (\% ) = (\Delta m/\rho )/V \times 100 \%$$

where Δ*m* was in representative of the difference in separators mass before and after impregnating in hexadecane, *ρ* stood for the density of hexadecane, and *V* implied the total volume of separators.

### Measurement of Thermal Shrinkages

The thermal shrinkages of MOF coated separators or PP separator were evaluated through Eq. (6):

6 $$Electrolyte retention\;\; (\% ) = (D_{2} - D_{1} )/D_{2} \times 100 \%$$

where *D*_*1*_ mean the diameters of separator after the heating, and *D*_*2*_ was on behalf of the diameters of the separator before the heating.

### Materials Characterizations

X–ray diffractometer (XRD) patterns were analyzed of as-synthesized materials by the powder X–ray diffractometer (D8–ADVANCE diffractometer, Germany), which was operated at a sweep rate of 10° min^−1^ and *2θ* scale ranging from 10 to 50. The morphologies of all samples were observed using field emission scanning electron microscopy (SEM, JSM–6701F, JEOL, Japan) and transmission electron microscopy (TEM, JEM–200, JEOL, Japan). Furthermore, the elemental mapping of samples was recorded utilizing an energy spectrometer (EDS) equipped with TEM equipment. The surface chemical compositions of the samples were confirmed by X–ray photoelectron spectroscopy (XPS, AXIS SUPRA, Shimadzu, Japan). The specific surface area and pore size distribution of samples were obtained by means of the Brunauer–Emmett–Teller (BET) and Saito–Foley (SF) methods, respectively. Fourier transform infrared spectrometer (FT–IR) measurements were conducted on a FT–IR spectrometer (IRXross, Japan). The Raman spectra were performed on a LabRAM HR Evolution Raman spectrophotometer (HORIBA Jobin Yvon S.A.S. France). The dynamic contact angle of separator was obtained by a contact angle instrument (SL250, China). The TGA measurements of samples and separators were performed on a TGA instrument (DTG–60, Shimadzu, *Japan*) between room temperature and 800 °C in N_2_ atmosphere with a ramp rate of 10 °C min^−1^.

### Density Functional Theory Calculations

All the density functional theory calculations (DFT) were performed using the dDMol3 module of materials studio (2020) package. The basis group general function was chosen as GGA/PW91 and the optimized molecules were all conducted with this basis group. The operations of calculations were then performed. The adsorption energy between polysulfides and MOF was calculated according to the following formula:

7 $$E_{ads} = E_{total} - E_{sub} - E_{adsor}$$

Among them, *E*_*total*_*, E*_*sub*_*,* and* E*_*adsor*_ represented the energy of the integrated material, the substrate, and the energy of adsorbed polysulfide molecules, respectively.

## Results and Discussion

**Fig. 1:**
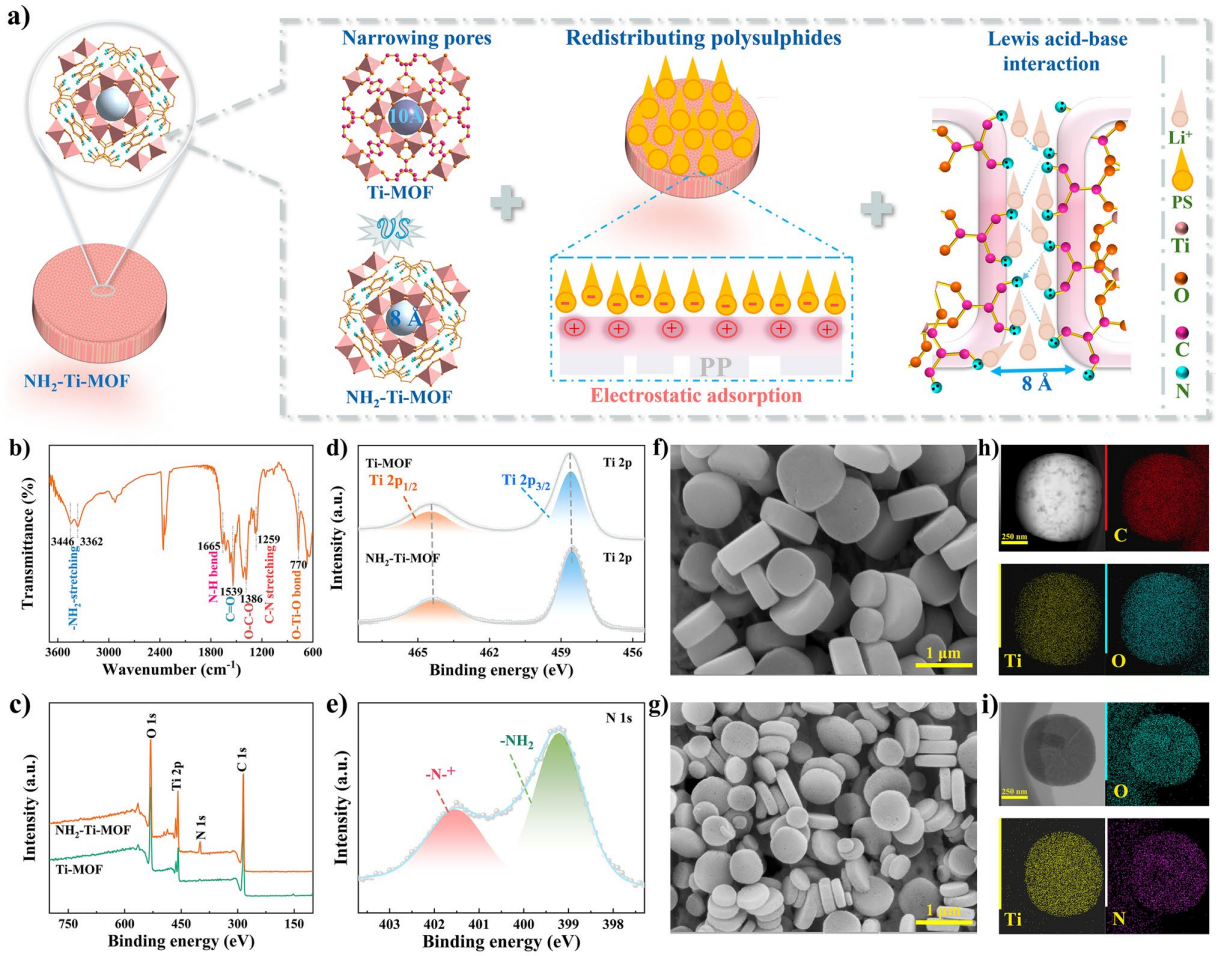
**a** Schematic illustration of roles performed by –NH_2_ in NH_2_–Ti–MOF on separator modification. **b** FT-IR spectrum of NH_2_–Ti–MOF. **c** XPS full spectra, high–resolution XPS spectra of **d** Ti 2*p* and **e** N 1*s*. **f** and **g** SEM images of Ti–MOF and NH_2_–Ti–MOF. **h, i** EDS mapping of Ti–MOF and NH_2_–Ti–MOF

As mentioned above, it is critical that the ion–selective channel is supposed to be around 0.8 nm to modify separator. Meanwhile, the Li^+^ migration in MOF pores and reuse of blocked polysulfides also need to be considered. Based on this, Ti–MOF is functionalized using the –NH_2_ group to meet the above requirements, due to its homogeneous microporous structure. Firstly, NH_2_–Ti–MOF is fabricated by a solvothermal approach. Afterward, it is subjected to a low–temperature under N_2_ atmosphere to remove residual solvent (Fig. S1), so the high porosity characteristics of NH_2_–Ti–MOF could be fully exerted. Thanks to the roles of –NH_2_, the pore size of MOF is narrowed from the original 0.95–1.1 to 0.83–0.86 nm after aminating, and this pore size could sequester thoroughly polysulfides with various chain lengths. Above all, the –NH_2_ group is able to adsorb polysulfides blocked by size effect to the surface of MOF with the help of electrostatic adsorption, facilitating a comparatively exhaustive electrochemical discharging of cathode. Polysulfides that absorbed on the MOF surface would return to cathode and engage in an electrochemical reaction during charging, enabling a great utilization of active materials. Besides, the Li^+^ migration would be facilitated through the formation of transfer channels driven by Lewis acid–base interaction between Li^+^ and –NH_2_ group in the sub-nanopore channels of NH_2_–Ti–MOF (Fig. 1a). Therefore, the Li–S batteries assembled with NH_2_–Ti–MOF modified separator exhibit a delightful behavior. For comparison, Ti–MOF is also used as separator modified layer, and it merely focuses on blocking the long–chain polysulfides on the cathode side using size–sieving effect.

FT-IR is carried out to verify the successful introduction of –NH_2_ group into Ti-MOF (Fig. 1b). The characteristic peaks at 3446 and 3362 cm^−1^ are attributed to the stretching of the –NH_2_, while the N–H bend vibration is observed at 1665 cm^−1^ [28]. In addition, the appearance of vibration peaks at 1539 and 1386 cm^−1^ are allocated to the C = O and C–O–C, respectively [29]. The stretching vibration peak of C–N and vibration bond of O–Ti–O are located at 1259 and 770 cm^−1^, respectively [30]. XPS is then applied to analyze the elemental components and chemical states of NH_2_–Ti–MOF and Ti–MOF. As suggested in Fig. 1c, the full XPS spectra reveal the characteristic peaks of C 1*s*, O 1*s*, and Ti 2*p* at 284, 531, and 458 eV, respectively. The Ti 2*p* spectra both of two samples could be deconvoluted into two peaks, corresponding to Ti 2*p*_1/2_ and Ti 2*p*_3/2_, respectively (Fig. 1d) [31, 32]. Moreover, the NH_2_–Ti–MOF displays N 1*s* spectrum at 399 eV, which is associated with –N–^+^ (≈401 eV) and –NH_2_ (399 eV), respectively (Fig. 1e) [33, 34]. The above results demonstrate the satisfactory fabrication of Ti–based MOF. SEM is a powerful technology that is usually used to observe the morphologies of materials. As depicted in Fig. 1f, g, both Ti–MOF and NH_2_–Ti–MOF present a typical cake–like morphology, and remarkably, the size of NH_2_–Ti–MOF is smaller than that of Ti–MOF, which primarily resulted from the molecular weight of 2–NH_2_ terephthalic acid is larger than that of terephthalic acid. Cake-like morphologies are a result of the combination of the coordination properties of titanium, the geometrical configuration of organic ligands, and the anisotropic growth induced by the synthesis conditions. Although amino modification decreases the pore size of Ti–MOF, it does not change the basic mode of crystal growth, and thus the two morphologies are similar [28, 29]. Concurrently, the TEM characterization findings testify to this observation (Figs. S2 and S3). Further, the high-resolution TEM images clarify the nanoscale porous structure of NH_2_–Ti–MOF and Ti-MOF (Fig. S4). Furthermore, the EDS mapping results indicate that the C, O, Ti elements and C, O, Ti, N elements are uniformly distributed in Ti-MOF and NH_2_–Ti–MOF, respectively (Fig. 1h, i).

**Fig. 2:**
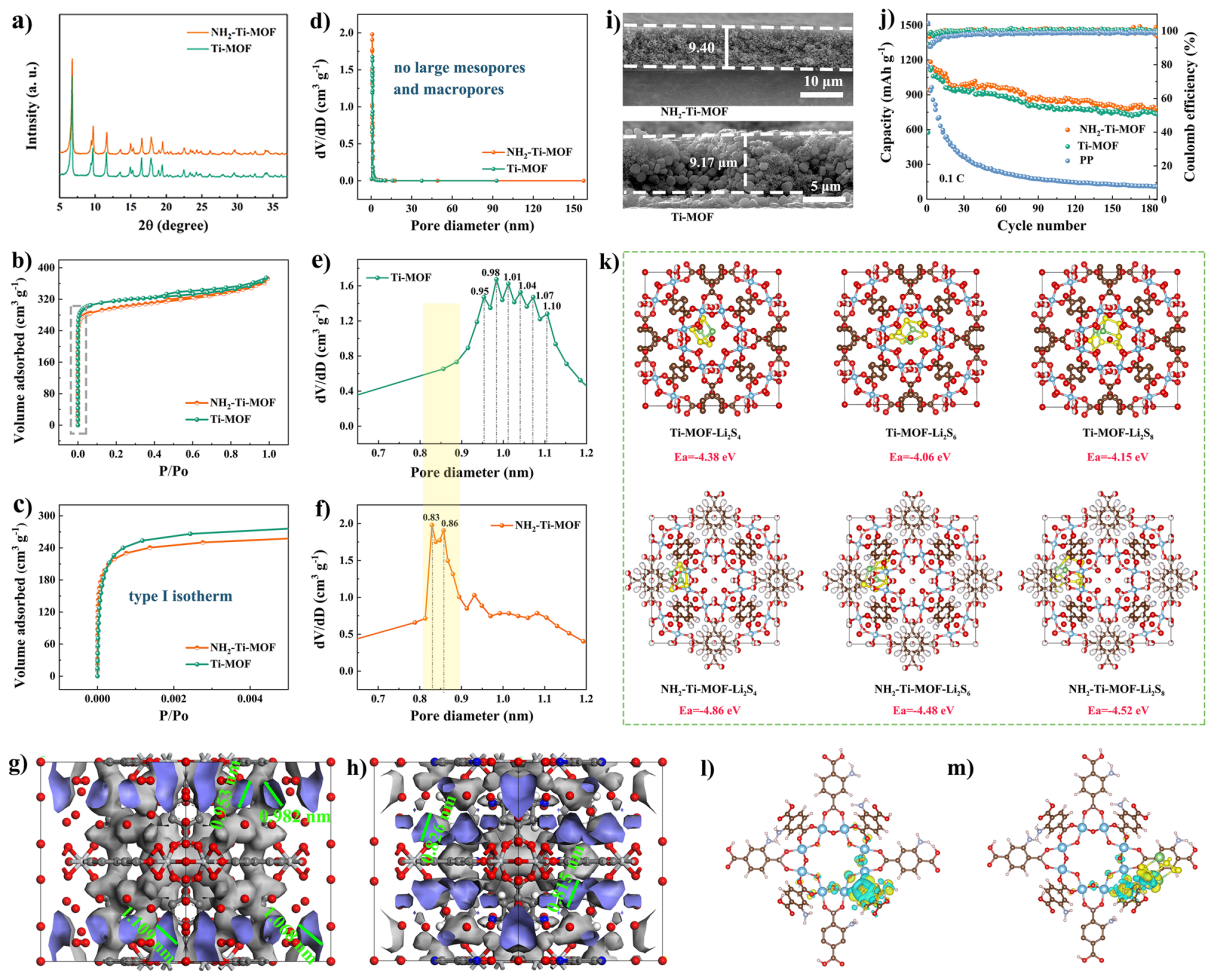
**a** XRD patterns. **b, c** N_2_ adsorption–desorption isotherms, **d** pore size distributions, **e, f** micropore size distributions of Ti–MOF and NH_2_–Ti–MOF. DFT theoretical simulation pores of **g** Ti–MOF and **h** NH_2_–Ti–MOF. **i** Cross-sectional SEM images of NH_2_–Ti–MOF and Ti–MOF modified separators. **j** Long cyclic performances at 0.1C with different separators. And **k** DFT calculations of the absorption energy of Ti–MOF and NH_2_–Ti–MOF toward various long–chain polysulfides (Li_2_S_4_, Li_2_S_6_, and Li_2_S_8_). Charge density difference plot **l** before and **m** after for Li_2_S_6_ interacting with NH_2_–Ti–MOF

XRD is conducted to characterize the two samples, and the crystal structures are consistent with those previously reported, which further confirms the satisfactory preparation of NH_2_–Ti–MOF and Ti–MOF. Besides, the XRD results also suggest that the low-temperature heat treatment would not damage the crystal structure of samples. Similarly, introduction of the –NH_2_ group has not altered the crystal structure of Ti–based MOF, thus ruling out the discrepancy in electrochemical properties arising from the crystal structure differences (Fig. 2a). The N_2_ adsorption–desorption is employed for testing the pore size distributions as well as specific surface areas. As presented in Fig. 2b and c, the isotherms of NH_2_–Ti–MOF and Ti–MOF reveal I-type isotherms without hysteresis loops, indicative of a typical microporous structure. The BET surface area of NH_2_–Ti–MOF and Ti–MOF are calculated to be 1168, and 1248 m^2^ g^−1^, respectively. Likewise, the pore distribution results are further confirmed that NH_2_–Ti–MOF and Ti-MOF only possess micropores (Fig. 2d).

Furthermore, the pore sizes of Ti–MOF are majorly distributed in 0.95– 1.1 nm, which is slightly larger than those of polysulfides partially, thereby it is somewhat unfavorable for the inhibition of polysulfides (Fig. 2e). On the contrary, after introduction the –NH_2_ group, pore sizes are narrowed to 0.83 and 0.86 nm with a fairly uniform distribution (Fig. 2f). And the pore size distributions of Ti–MOF and NH_2_–Ti–MOF were further calculated by DFT model, which is in agreement with the experimental results (Fig. 2g, h). Such pore sizes enable NH_2_–Ti–MOF to enjoy much higher ion selectivity by virtue of size–sieving effect. The cross–sectional SEM images display that NH_2_–Ti–MOF and Ti–MOF coatings are homogeneous and dense with a mere thickness around 9 μm (Fig. 2i). In view of the triple roles of –NH_2_ group, such as size–sieving effect, electrostatic adsorption, and Lewis acid–base interaction, Li–S batteries assembled with NH_2_–Ti–MOF modified separator demonstrate enhanced cycle life at 0.1C compared to Ti–MOF modified separator, let along PP separator (Fig. 2j). Moreover, density functional theory (DFT) calculations are employed to prove the –NH_2_ provides a stronger adsorption effect for clogged polysulfides. As depicted in Fig. 2k, the surface adsorption energy of Li_2_S_4_, Li_2_S_6_, and Li_2_S_8_ adsorbed on NH_2_–Ti–MOF surface (− 4.86, − 4.48, and − 4.52 eV) are considerably higher than on Ti–MOF (− 4.38, − 4.06, and − 4.15 eV). In addition, charge density difference is one of the crucial approaches to study the electronic structure, which allows to visually analyze the electronic changes after materials interact with the other. In Fig. 2l, m, the yellow area represents electron aggregation and the green area represents electron reduction. It can be observed that the electrons of groups on the structure of NH_2_–Ti–MOF are transferred from the –NH_2_ group to the Li_2_S_6_ molecule after NH_2_–Ti–MOF adsorbs Li_2_S_6_. Such results further prove the adsorption of polysulfides on NH_2_–Ti–MOF. Notably, for the purpose of demonstrating the sieving effect of NH_2_–Ti–MOF’ 0.83 nm pore size on polysulfides, we simulated and calculated the diffusion energy barrier of S_6_^2−^ through pores using Molecular Dynamics, as calculated, the transport energy barrier of S_6_^2−^ is upped to 150.8 kJ mol^−1^ (Fig. S5). The large energy barrier demonstrates that S_6_^2−^ is very difficult to drive through 0.83 nm pore size, indicating that 0.83 nm pore channel can effectively sieve polysulfides and Li^+^ using size-sieving effect. Based on the above-mentioned benefits, the NH_2_–Ti–MOF coated functionalized separator could be superior in Li–S batteries, such as sieving polysulfides, facilitating Li^+^ migration, as well as coordinating the redistribution and reuse of blocked polysulfides.

**Fig. 3:**
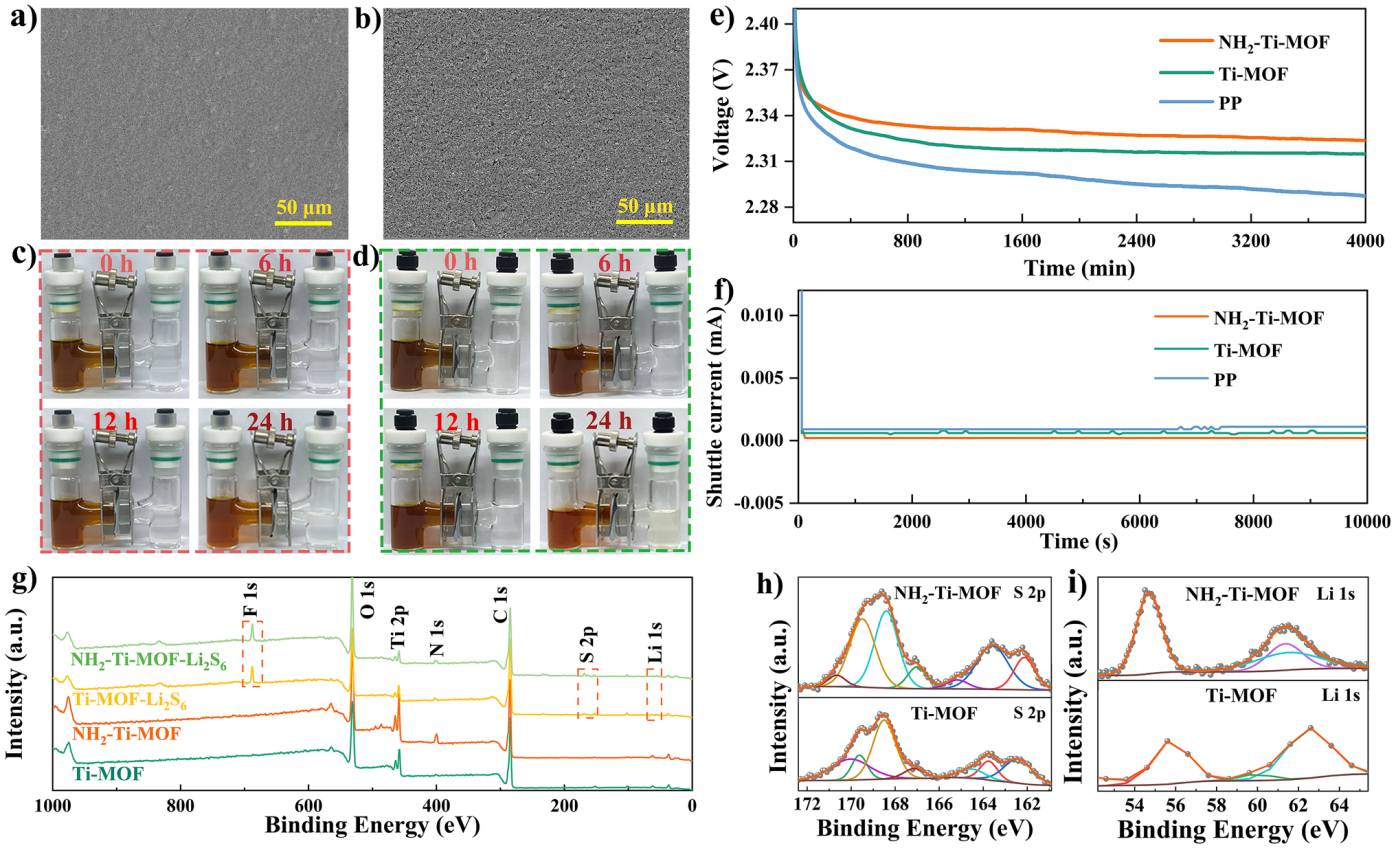
Top-surface SEM images and H–type Li_2_S_6_–permeation experiments of **a, c** NH_2_–Ti–MOF and **b, d** Ti–MOF modified separators. **e** Self–discharging behavior and **f** shuttle current of pristine PP separator, NH_2_–Ti–MOF, and Ti–MOF modified separators. **g** XPS spectra of NH_2_–Ti–MOF and Ti–MOF modified separators before and after Li_2_S_6_–permeation tests, and **h** high–resolution XPS spectra of S 2*p* and **i** Li 1*s*

From Fig. 3a and b, it is could observe that the surface of PP separator becomes even, dense, and smooth, respectively, after coating NH_2_–Ti–MOF and Ti–MOF, which completely covers the reticulation structure composed of larger pores (tens of micrometers) in PP separator [16]. In this way, NH_2_–Ti–MOF efficiently prevents the migration of polysulfides to anode and realizes the redistribution of polysulfides by virtue of its 0.83 nm pore size and electrostatic adsorption. Simultaneously, Li^+^ could move between anode and cathode completely unimpeded. Aiming to examine the block effect of NH_2_–Ti–MOF modified separator on polysulfides shuttle, a visual permeation test is performed. In H–type vessel, the left and right chambers contain Li_2_S_6_ solution and pure solvent liquid, respectively, which are separated by modified separators to evaluate their ability of suppressing polysulfides diffusion via the color change of solution in the right chamber. Figure 3c devices that the right chamber solution maintains a transparent color after up to 24 h, indicative of the absence of significant polysulfides crossover, benefiting from an excellent screening ability for polysulfides of NH_2_–Ti–MOF modified separator. In contrast, the color of solution in the right chamber in Fig. 3d changes to a faint light–yellow after 24 h, which indicates that Ti–MOF could block polysulfides shuttle but not completely, and thus causing a slight crossover of polysulfides.

Meanwhile, the self–discharging and shuttle current tests can also be conducted to indirectly detect polysulfides shuttling. The open–circuit voltage of batteries assembled with NH_2_–Ti–MOF modified separator decreases from 2.78 to 2.32 V during the test period, while Ti–MOF modified separator and pure PP separator fall from 2.78 to 2.31 V and 2.78 to 2.29 V, respectively, during the same testing period (Fig. 3e). The self–discharging of Li–S batteries is largely induced by the reaction between shuttled polysulfides and Li anode. The above results show that NH_2_–Ti–MOF possesses a highly efficient ability to inhibit polysulfides shuttle, which is in accordance with the results of polysulfides shuttle experiments. Figure 3f manifests that the batteries with NH_2_–Ti–MOF modified separator possesses a smaller shuttle current than that with Ti–MOF modified separator and pure PP separator. Moreover, XPS technique is employed to detect polysulfides on the NH_2_–Ti–MOF and Ti–MOF modified separators after Li_2_S_6_ permeation experiments. Signature peaks of S and Li recognized on the both modified separator surfaces, and peaks intensity on the NH_2_–Ti–MOF modified separator surface is sharper, suggesting that NH_2_–Ti–MOF is more superior in the adsorption of polysulfides (Fig. 3g–i). This is because NH_2_–Ti–MOF could inhibit the diffusion of polysulfides and absorb them by virtue of size effect and electrostatic absorption, respectively, whereas Ti–MOF owns merely size effect.

**Fig. 4:**
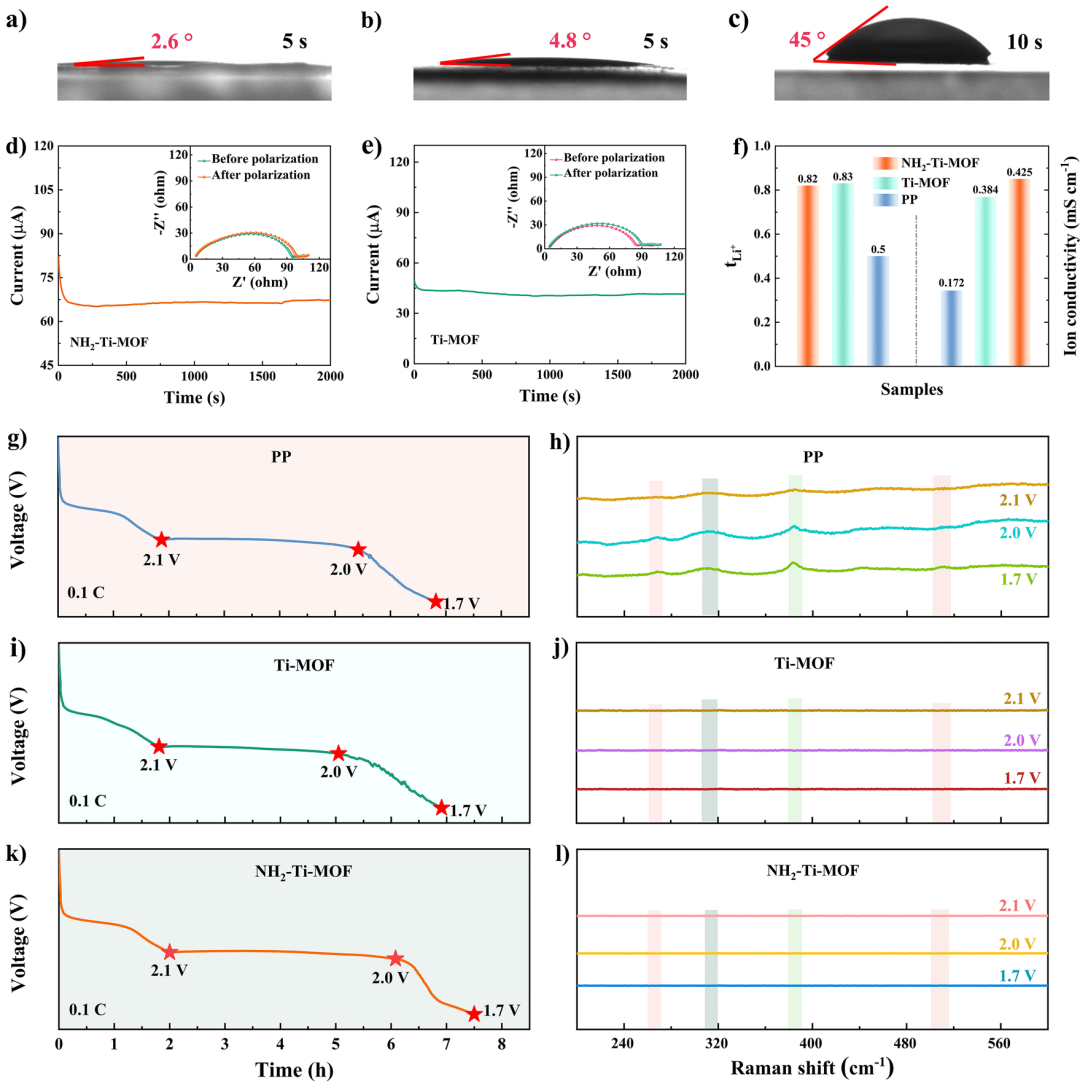
Electrolyte dynamic contact angles of **a** NH_2_–Ti–MOF modified separator, **b** Ti–MOF modified separator, and **c** PP separator. Time–dependence curves of polarization for batteries with **d, e** NH_2_–Ti–MOF and Ti–MOF modified separators (insets display the corresponding EIS before and after polarization), **f** Li^+^ migration numbers and ion conductivities. Discharging profile and Raman spectra close to Li anode after differing voltages discharging of Li–S batteries with **g, h** PP separator; **i, j** Ti–MOF modified separator; **k, l** NH_2_–Ti–MOF modified separator

The superior specific surface area and plentiful porosity of Ti–based MOF considerably enlarge the contact area between functional separators and electrolytes. Also, the polar of Ti–based MOF grants modified separators with a much better affinity for electrolyte, advancing the wettability and permeability, especially the introduction of polar functional group –NH_2_ in MOF. Most of all, the porous feature of Ti–based MOF coatings confers capillary forces that enhance the adhesion of electrolyte. These virtues can greatly guarantee Li^+^ transport so as to reduce batteries polarization. As highlighted in Fig. 4a–c, compared with pure PP separator, NH_2_–Ti–MOF and Ti–MOF modified separators present remarkable improvement for electrolyte wettability as expected. The dynamic contact angles of electrolyte on NH_2_–Ti–MOF and Ti–MOF modified separator surfaces are obviously smaller than that of pure PP separator. In addition, the testing results of electrolyte uptake, retention rate, and porosity (Figs. S6–S8) reveal that NH_2_–Ti–MOF and Ti–MOF modified separators also present satisfactorily. Batteries are quite likely to be operated at high temperatures due to extreme weather, which may potentially make separator unstable and impair the storage performance of batteries. Therefore, the thermal stability of several separators is inspected at varying heating temperatures (Figs. S9–S11). Obviously, PP separator suffers a pronounced shrinkage compared to NH_2_–Ti–MOF and Ti–MOF modified separators at 180 °C, while the NH_2_–Ti–MOF coating still retains its structural completeness and results in a “self–supporting” membrane, even when the PP layer is fused.

The migration of other ions across the separator will be affected when the shuttling of polysulfides is inhibited via size effect of MOF modification layer. Li^+^ migration behaviors in NH_2_–Ti–MOF and Ti–MOF modified separators are further investigated quantitatively. Specifically, the Li^+^ transference numbers of NH_2_–Ti–MOF and Ti–MOF modified separators are determined to be 0.82 and 0.83, respectively, which is preferred to pure PP separator (Fig. 4d–f). Although the pore size of NH_2_–Ti–MOF is smaller than that of Ti–MOF, the Li^+^ migration number of NH_2_–Ti–MOF modified separator is comparable to that of Ti–MOF modified separator. This is mainly because the nitrogen atom in –NH_2_ has a lone pair of electrons, which can act as a Lewis base to coordinate with Li^+^ to form Li^+^–N weak coordination bond. Such coordination can help to reduce the solvation energy of Li^+^ and accelerate its desolvation process, thereby accelerating the migration of Li^+^ from electrolyte to separator interface. Especially in the ether electrolyte, the polarity of –NH_2_ provides a stronger attraction to Li^+^. Moreover, this effect provides temporary “conductive paths” that can guide Li^+^ transport in a directional manner. The polar character of –NH_2_ forms a local electric field in MOF pores, which directs Li^+^ to diffuse rapidly along a given path by electrostatic attraction and reduces the energy loss caused by disordered migration. The above results suggest that NH_2_–Ti–MOF and Ti–MOF modified separators, especially the former, could reduce the circuitous route of Li^+^ diffusion and are favorable for improving the screening of ions. Besides, the NH_2_–Ti–MOF modified separator exhibits a higher ionic conductivity of 0.425 mS cm^−1^ than that of Ti–MOF modified separator (0.384 mS cm^−1^) and PP separator (0.172 mS cm^−1^), respectively (Figs. 4f and S12). The effectiveness of diverse separators in inhibiting polysulfides shuttle is probed by *ex–situ* Raman spectroscopy. When the discharging voltage of batteries reaches 2.1, 2.0, and 1.7 V, respectively, the Raman signals are captured from the separator surfaces oriented to the lithium anode. Wherein, there are a series of evident peaks that appeared for pure PP separator at varied voltages, indicative of heavily polysulfides shuttle, which is very unfavorable for Li–S batteries (Fig. 4g and h). Whereas, it is gratifying to note that NH_2_–Ti–MOF and Ti-MOF modified separators deliver no polysulfides’ signals at the same testing conditions, signifying that polysulfides shuttle can be entirely restrained (Fig. 4i–l). The aforementioned results manifest that NH_2_–Ti–MOF modified separators could not only prevent polysulfides shuttle but also boost Li^+^ migration. Concretely, –NH_2_ not only enhances the utilization of active materials via electrostatic adsorption of polysulfides but also provides a Li^+^ transfer channel through Lewis acid–base interaction with Li^+^. Such brilliant separator guarantees the outstanding electrochemical performance of Li–S batteries.

**Fig. 5:**
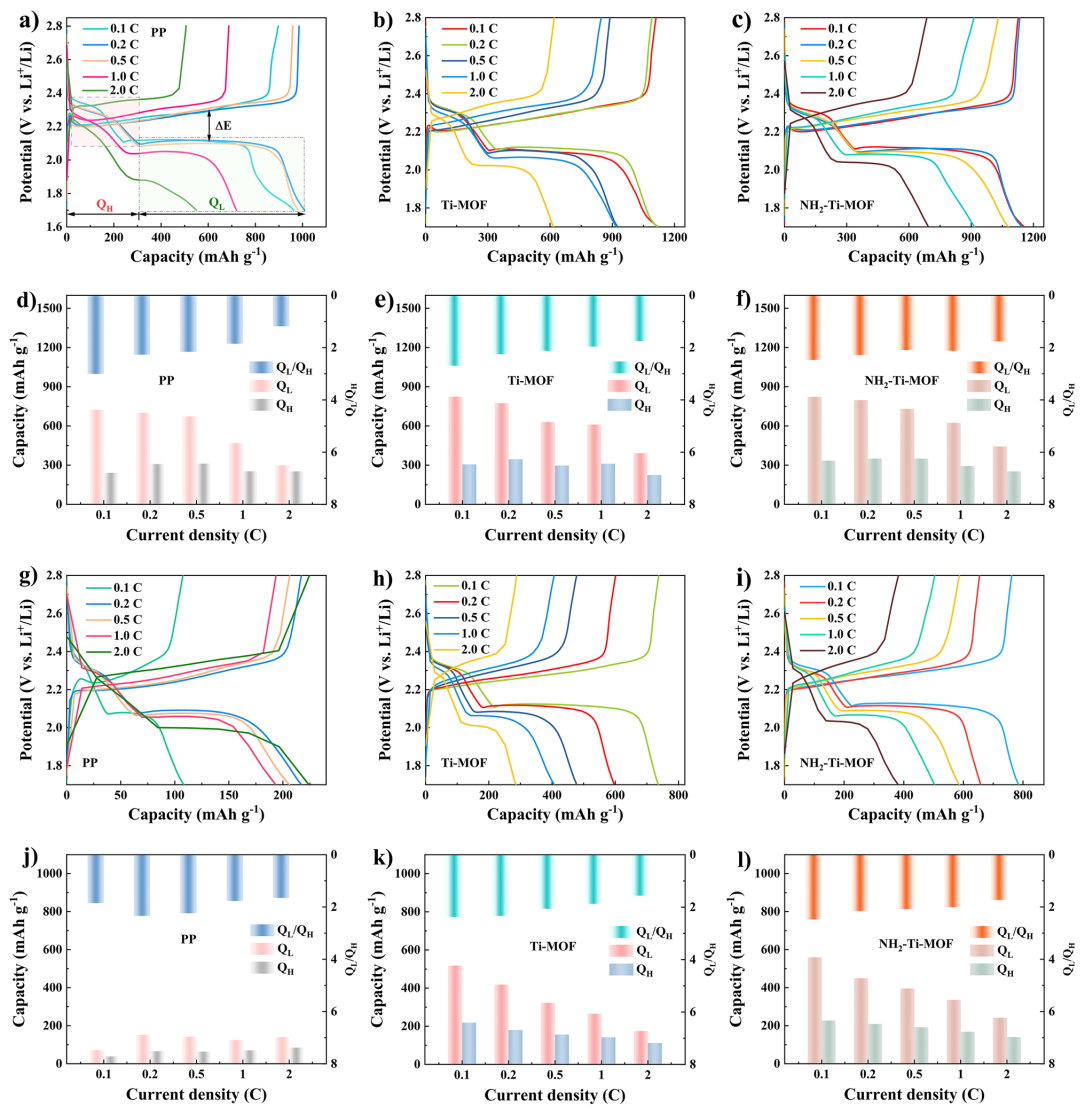
Galvanostatic charging/discharging curves at diverse current rates of Li–S batteries before cycling and the corresponding values of *Q*_L_, *Q*_H_, and *Q*_L_/*Q*_H_ using **a** and **d** PP separator, **b, e** Ti–MOF modified separator, **c, f** NH_2_–Ti–MOF modified separator. Galvanostatic charging/discharging curves after cycling at diverse current rates and the corresponding values of *Q*_L_, *Q*_H_, and *Q*_L_/*Q*_H_ of using **g, j** PP separator; **h, k** Ti–MOF modified separator; **i, l** NH_2_–Ti–MOF modified separator

To assess the promising inhibition of polysulfides shuttle with NH_2_–Ti–MOF and Ti–MOF modified separators, the electrochemical behaviors of Li–S batteries assembled with them as separators are investigated. Firstly, the cycling stability of Li–S batteries with activated and inactivated MOF modified separators are compared at 0.1C, and the former is superior (Fig. S13). This may be the activated MOF exposes higher porosity and active sites after removing the residual solvents. Accordingly, the activated NH_2_–Ti–MOF and Ti–MOF are adopted for the investigation in this work. The GCD profiles of Li–S batteries with various separators are illustrated in Fig. 5a–c (all batteries are subjected to activate for 3 cycles at 0.1C). There are two representative discharging plateaus and a charging plateau in all GCD profiles. Wherein, *Q*_H_ represents the specific capacity for the electrochemical reduction of sulfur to long–chain polysulfides (light pink area in Fig. 5a), and *Q*_L_ stands for the specific capacity for the electrochemical reduction of long-chain polysulfides to Li_2_S_2_ or Li_2_S (light green area in Fig. 5a) [35–37]. Notably, the values of *Q*_L_ and *Q*_H_ suggest the completion degree of cathode active substance reduction reaction, correlated with the rejection of shuttle effect and the complete conversion of solid polysulfides. In detail, given that there are 4 electrons at high discharging plateau and 12 electrons at low discharging plateau, the logical *Q*_L_/*Q*_H_ ratio is supposed to be 3. Yet, in reality, this is not the case.

After 3 cycles of activation at a low current rate, the first GCD profiles at any current rates reveals that the *Q*_H_ and *Q*_L_ values of Li–S batteries with NH_2_–Ti–MOF modified separator outperform those of Ti–MOF modified separator at any, let alone relative to pure PP separator (Fig. 5d–f). Specifically, the values of *Q*_H_ and *Q*_L_ for NH_2_–Ti–MOF modified separator are 349.7 and 796.7 mAh g^−1^ at 0.2C, respectively, while Ti–MOF modified separator and bare PP separators are 344.4 and 773.2 mAh g^−1^ and 307.9 and 700.2 mAh g^−1^, respectively. More importantly, the NH_2_–Ti–MOF modified separator displays a higher *Q*_L_/*Q*_H_ value (2.28) than Ti–MOF modified separator (2.25) and PP separator (2.27). Accordingly, compared with the pores of pure PP which exert no inhibition on polysulfides shuttle, NH_2_–Ti–MOF and Ti–MOF modified separators firstly restrain polysulfides migration to anode by means of size–sieving effect. As the current rate increases, NH_2_–Ti–MOF modified separator will adsorb the captured polysulfides on surface, fully exposing the active material within cathode to continue discharge without obstruction. Unlike, Ti–MOF modified separator is incapable of assisting in the redistribution of polysulfides, leading them cover the surface of cathode, which in turns prevents active material contact with Li^+^, thereby degrading the batteries’ performance at high current rates. This behavior will become more obvious after batteries have gone through long cycling.

Figure 5g–i illustrates the GCD curves of Li–S batteries with diverse separators after long–term cycling. It is apparent that the specific capacity of pure PP separator drops the most, followed by Ti–MOF modified separator, and the least is NH_2_–Ti–MOF modified separator. The time of second stage discharging plateau in the batteries containing NH_2_–Ti–MOF modified separator displays a considerable retention after long cycles, indicative of improved sulfur utilization and conversion in the interconversion process of Li_2_S_4_ to Li_2_S. Such advantageous specific capacity is inseparable from the size-sieving effect of NH_2_–Ti–MOF and the adsorption effect of the –NH_2_ on polysulfides. Furthermore, the *Q*_H_ and *Q*_L_ of batteries assembled with three different separators undergo considerable discrepancy after long cycling (Fig. 5j–l). As expected, the Li–S batteries with NH_2_–Ti–MOF modified separator still retain the highest specific capacity for *Q*_H_ and *Q*_L_ compared with Ti–MOF modified separator and pristine PP separator at varied current rates, such as at 0.5C, NH_2_–Ti–MOF: *Q*_H_: 208.6 mAh g^−1^, *Q*_L_: 449.5 mAh g^−1^; Ti–MOF: *Q*_H_: 179.5 mAh g^−1^, *Q*_L_: 417.9 mAh g^−1^; PP: *Q*_H_: 65 mAh g^−1^, *Q*_L_: 152 mAh g^−1^. This further demonstrates that NH_2_–Ti–MOF could inhibit polysulfides shuttle and redistribute blocked polysulfides, which is conducive to the realization of excellent electrochemical properties.

**Fig. 6:**
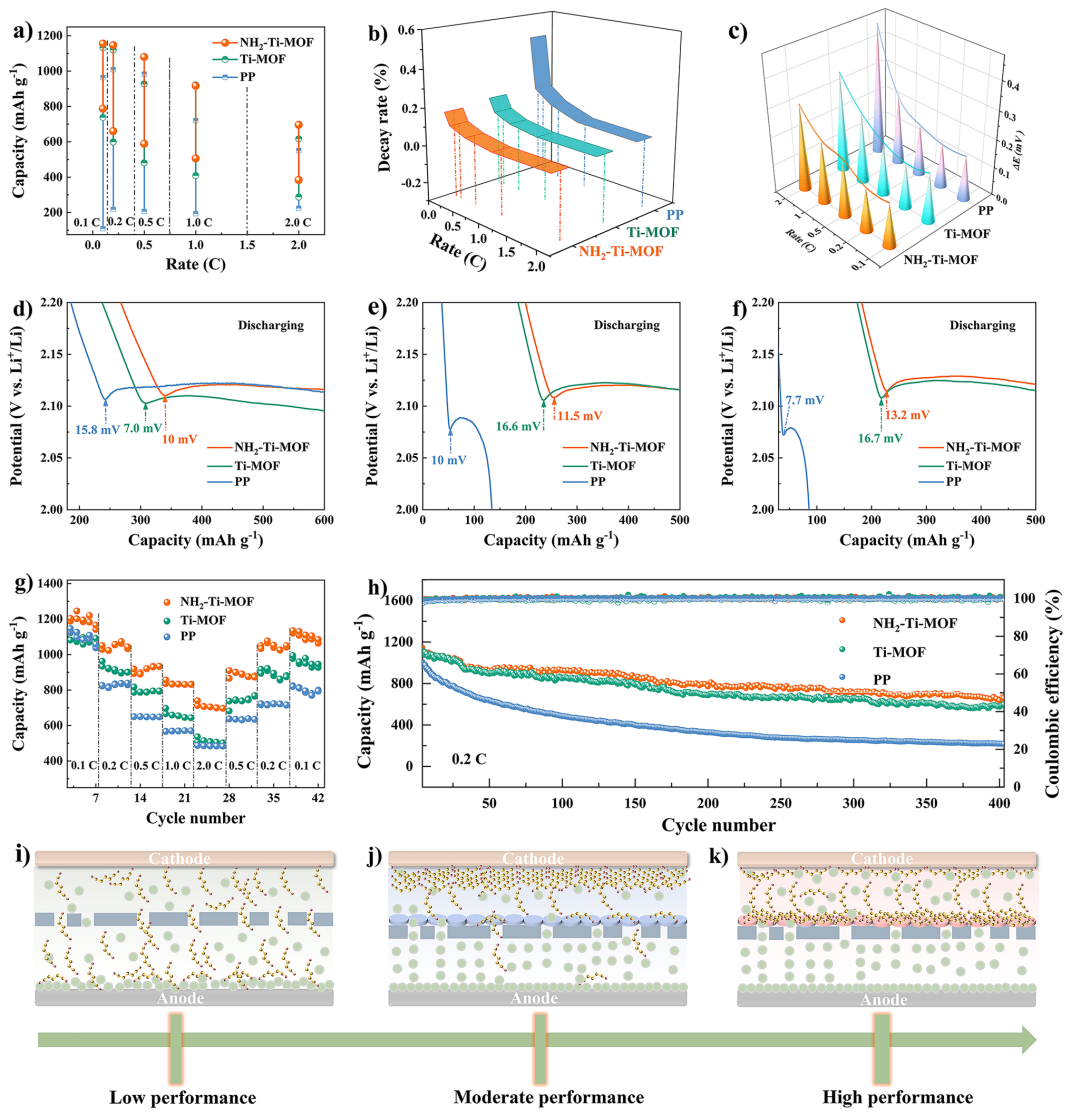
Electrochemical behaviors of Li–S batteries with various separators: **a** Specific capacity differences at diverse current rates before and after cycling. **b** Capacity fading rates at varied current rates. **c** ΔE acquired from charging/discharging profiles. Voltage polarization of Li_2_S deposition at 0.1C of **d** on initial cycles, **e** over 100 cycles, and **f** over 186 cycles. **g** Rate performances at distinct current rates. **h** Long cyclic performances at 0.2C. **i-k** Performances failure mechanism of Li–S batteries assembled with different separators

The coin batteries incorporating NH_2_–Ti–MOF modified separator manifest a promising specific capacity outperforming those of PP and Ti–MOF modified separators before and after cycles, as seen in Fig. 6a. As well, the capacity fading rates follow the same regularity (Fig. 6b, and Table S1). Moreover, the influence of polysulfides on cathode is also reflected indirectly in the polarization of batteries. The electrochemical polarization of batteries containing NH_2_–Ti–MOF modified separator, Ti–MOF modified separator, and PP separator are investigated and compared as exhibited in Fig. 6c. The batteries with NH_2_–Ti–MOF modified separator present the lowest polarization voltage (Δ*E*) (0.15 V) compared with Ti–MOF modified separator (0.173 V) and PP separator (0.16 V) at 0.1C. And this phenomenon is increasingly salient with rising current rates (Table S2). Obviously, polysulfides covered on the cathode surface will make reactive material contact with Li^+^ more challenging, which can be avoided by adsorbing polysulfides on the separator surface. What is more, the batteries with NH_2_–Ti–MOF and Ti–MOF modified separators display a moderate Li_2_S nucleation barrier than PP separator at 0.1C (Fig. 6d). With accumulating of cycles, the Li_2_S nucleation barrier of NH_2_–Ti–MOF modified separator remains almost invariant, while Ti–MOF modified separator increases abruptly (Fig. 6e, f). It should be noted that the Li_2_S nucleation barrier of pure PP separator gets lower with increasing cycles, which might be attributed to the absence of excess polysulfides on the cathode surface.

Undeniably, batteries assembled with NH_2_–Ti–MOF modified separator express admirable rate performance relying on size-sieving effect and electrostatic adsorption at varied current rates. Especially, even returning to the initial 0.1C, an inspiring capacity of 1134.8 mAh g^−1^ along with a higher capacity retention of 94.2% is achieved, over that for the Ti–MOF modified separator (994.9 mAh g^−1^, 88.5%), and PP separator (820.3 mAh g^−1^, 71.5%) (Fig. 6g). Expectedly, the long–cycle behaviors at 0.2C certify that batteries with NH_2_–Ti–MOF modified separator could deliver a rosy capacity of 658.1 mAh g^−1^ after 400 cycles, with a nether more capacity fading ratio of 0.106% per cycle (Fig. 6h). In contrast, Ti–MOF modified separator and PP separator display an impecunious capacity of 597.4 and 217.1 mAh g^−1^, with a faster capacity fading ratio of 0.12% and 0.2% at the same condition, respectively. In light of the above results, pure PP separator is powerless against the migration of polysulfides (Fig. 6i), and MOF with uniform and appropriate pore size distribution could hinder polysulfides from crossing PP separator pores with the help of the size effect. Yet, the congestion of polysulfides on the surface will exert an adverse effect on cathode, discouraging deeper discharging behaviors (Fig. 6j). Luckily, well–arranged adsorption of blocked polysulfides on separator surface site with the assistance of –NH_2_ groups to provide sufficient reaction space for the cathode actives could promote the utilization of the actives (Fig. 6k).

**Fig. 7:**
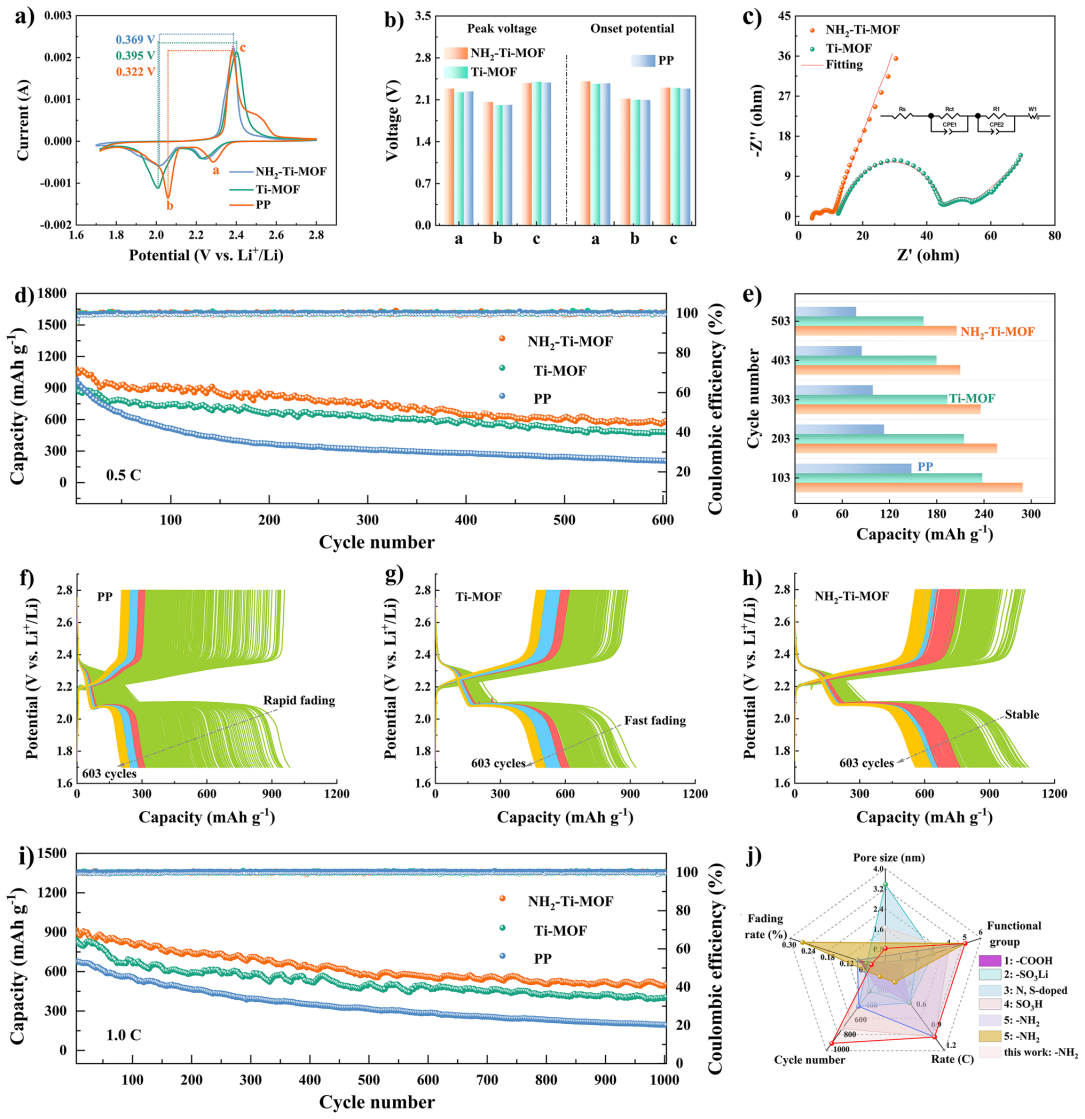
Electrochemical behaviors of Li–S batteries assembled with various separators: **a, b** CV profiles at 0.1 mV s^−1^ and corresponding peak voltages and onset potentials. **c** EIS spectra. **d** Long cyclic behaviors at 0.5C over 600 cycles, and **e** corresponding high plateau capacities at varying cyclic periodicities. Charging/discharging curves of **f** pure PP separator, **g** Ti–MOF modified separator, and **h** NH_2_–Ti–MOF modified separator over 600 cycles at 0.5C. **i** Long cycle life tests at 1.0C. **j** Performance comparison with other reported separators Li–S batteries using different functional groups and pore size

Figure 7a compares CV profiles of batteries with varied separators at 0.1 mV s^−1^. Specifically, for the NH_2_–Ti–MOF modified separator, the cathodic voltage peaks (a and b) behave far more positively, while the anodic voltage peak (c) behaves far more negatively, in potential than Ti–MOF modified separator and PP separator. The voltage difference between peak b and c of batteries with NH_2_–Ti–MOF modified separator is 0.322 V, which is far smaller than that of Ti–MOF modified separator (0.395 V) and PP separator (0.369 V), respectively, revealing that batteries based on NH_2_–Ti–MOF modified separator possesses a smaller polarization. Notably, the voltage difference of Ti–MOF modified separator is even higher than pure PP separator, indicating that the unarranged and trapped polysulfides do affect the cathodic reaction. Also, compared with Ti–MOF modified separator and PP separator, the batteries with NH_2_–Ti–MOF modified separator own a moderately higher peak potential and onset potential for reduction peaks (Fig. 7b). Incidentally, batteries with NH_2_–Ti–MOF modified separator display the highest peak current as shown in Fig. S14, indicative of satisfactory sulfur utilization. The EIS spectra (Figs. 7c and S15) are employed to verify the resistance of batteries with distinct separators. The results uncover that batteries based on NH_2_–Ti–MOF modified separator present the smallest charge transfer resistance (*R*_ct_) [38–40]. After fitting to the equivalent circuit (inset of Figs. 7c and S15), a smaller *R*_ct_ around 2.05 Ω is obtained, which is superior to Ti–MOF modified separator (32.7 Ω), and PP separator (463.9 Ω). The diffusion rates of Li^+^ are also assessed. As present in Fig. S16a, b, the slope of oxidation peak c for NH_2_–Ti–MOF modified separator is 0.208, which is appreciably higher than Ti–MOF (0.178). The higher Li^+^ diffusion coefficients during charging process proves our idea. It is also demonstrated that NH_2_–Ti–MOF could promote Li^+^ diffusion using Warburg impedance coefficients based on results of EIS simulations (Fig. S16c).

In addition, the cycling behavior over 600 cycles at 0.5C indicates that batteries with NH_2_–Ti–MOF modified separator emerge a graceful initial capacity of 1, 079.8 mAh g^−1^ and reserve a reversible capacity of 586.5 mAh g^−1^, accompanying a low fading rate of 0.07% per cycle and a favorable coulombic efficiency of about 100% (Fig. 7d). In contrast, the Ti–MOF modified separator and PP separator exhibit inferior capacities and rapid decay during the same cycling, fading from 925.9 to 478.6 mAh g^−1^ and from 982.8 to 206 mAh g^−1^, respectively, indicative of underutilization of polysulfides. Meanwhile, for high plateau capacities, batteries assembled with NH_2_–Ti–MOF modified separator yield a lower fade rate than Ti–MOF modified separator and pure PP separator under varying cycles (Fig. 7e). This reveals that NH_2_–Ti–MOF modified separator possesses favored inhibition of polysulfides shuttle while not interfering with the normal discharging of cathode. Similarly, the GCD curves with varying numbers of cycling cycles reinforce the superiority of NH_2_–Ti–MOF in Li–S batteries separator modification (Fig. 7f–h). Figure 7i is the long–term cyclic lifespan of batteries with different separators explored at 1.0C. Expectedly, the batteries with NH_2_–Ti–MOF modified separator deliver a promising initial capacity of 915.6 mAh g^−1^ and reserves 503.3 mAh g^−1^ after 1000 cycles with a low degradation rate of 0.045% per cycle. Whereas, the Ti-MOF modified separator and PP separator possess an unpleasant post–cycle capacity of 407.2 and 193.3 mAh g^−1^ with penniless fading rates of 0.056% and 0.073% per cycle, respectively. This result implies that the NH_2_–Ti–MOF modified separator is favorable for prolonged use of batteries, as also confirmed by the cycling capability after 1000 cycles at 2.0C, as shown in Fig. S17. Additionally, such low capacity attenuation of NH_2_–Ti–MOF modified separator is far superior to previously reported separator modifications using size effect and group modification (Fig. 7j) [23–25, 41–43]. Meanwhile, we disassembled batteries that were cycled for 1003 cycles at 1.0C and measured the morphologies of MOF. From Fig. S18, it can be clearly observed that the morphologies of NH_2_–Ti–MOF and Ti–MOF still maintain the cake-like shape and do not exhibit visible structural collapse. Further, the mapping images display the Ti, C, O, S and Ti, C, O, S, N elements on the surfaces of Ti–MOF and NH_2_–Ti–MOF modified layers. The results reveal that the structures of NH_2_–Ti–MOF and Ti–MOF are relatively stable. Eventually, the high S–loaded cathode is ready to investigate the practical application of NH_2_–Ti–MOF modified separator (Fig. S19). The capacities of thick electrodes with loadings of 2.1 and 3.0 mg could retain 525.1 and 403.7 mAh g^−1^ after 200 and 153 cycles at 0.2C, respectively, signifying that NH_2_–Ti–MOF modified separator performs nicely in inhibiting polysulfides shuttle for practical applications. So, the 0.83 nm pore size with electropositive –NH_2_ in separator interfacial layers is the most suitable for inhibiting polysulfides (Fig. S20).

## Conclusions

In summary, a NH_2_–Ti–MOF with sub-nanometer channel size is elaborately engineered and employed for Li–S batteries separator modification layer, aiming to address polysulfides shuttles. Benefitting from –NH_2_ in NH_2_–Ti–MOF, the modified separator offers multiple merits in reaching optimal electrochemical performance for Li–S batteries. Firstly, the introduction of -NH_2_ in sub-nanochannel narrows the pore size of Ti–MOF, enabling more precise control of Li^+^ and polysulfides migration by virtue of size-sieving effects. Further, the positively charged –NH_2_ adsorbs the negatively charged polysulfides via robust electrostatic interactions, leaving trapped polysulfides to stand on the separator surface in a well–organized manner. Consequently, when the NH_2_–Ti–MOF modified separator is employed in Li–S batteries, the batteries enjoy superior cycling stability with an ultralow fading rate of 0.07% per cycle over 600 cycles at 0.5C. Furthermore, an impressive initial capacity of up to 915.6 mAh g^−1^ at 1.0C is achieved and a final capacity of 503.3 mAh g^−1^ is maintained after 1000 cycles. This work demonstrates that electrostatic adsorption and Lewis acid–base interactions are expected to make up for the lack of size effects in inhibiting polysulfides shuttle, which is a promising approach to completely restrain polysulfides shuttle.

## Supplementary Information

Below is the link to the electronic supplementary material.

Supplementary file1 (DOCX 22871 KB)
