# Supplementary material for: Designing Amino Functionalized Titanium-Organic Framework on Separators Toward Sieving and Redistribution of Polysulfides in Lithium-Sulfur Batteries
Source: Nanomicro Lett. 2025 May 26;17:277. doi: 10.1007/s40820-025-01733-0 (PMC12106290; doi:10.1007/s40820-025-01733-0)
Supplement: Supplementary file 1 — Supplementary file1 (DOCX 22871 KB) [file 40820_2025_1733_MOESM1_ESM.docx]

Supporting Information for

**Designing Amino Functionalized Titanium-Organic Framework on Separators Toward Sieving and Redistribution of Polysulfides in Lithium-Sulfur Batteries**

Xiaoya Kang^1^, Tianqi He^1^, Hao Dang^1^, Xiangye Li^1^, YumengWang^1^, Fuliang Zhu^1^, Fen Ran^1^***

^1^State Key Laboratory of Advanced Processing and Recycling of Non-ferrous Metals, School of Materials Science and Engineering, Lanzhou University of Technology, Lanzhou 730050, P. R. China

***Corresponding author. E-mail: [ranfen@lut.edu.cn](mailto:ranfen@lut.edu.cn) or [ranfen@163.com](mailto:ranfen@163.com) (Fen Ran)

**Supplemenary Figures and Tables**

**
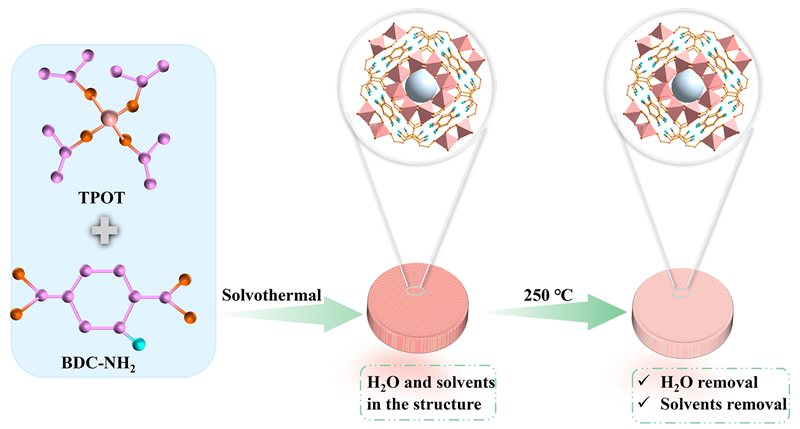
**

**Fig. S1** The preparation processes of NH_2_-Ti-MOF via the solvothermal method and followed by thermal calcination


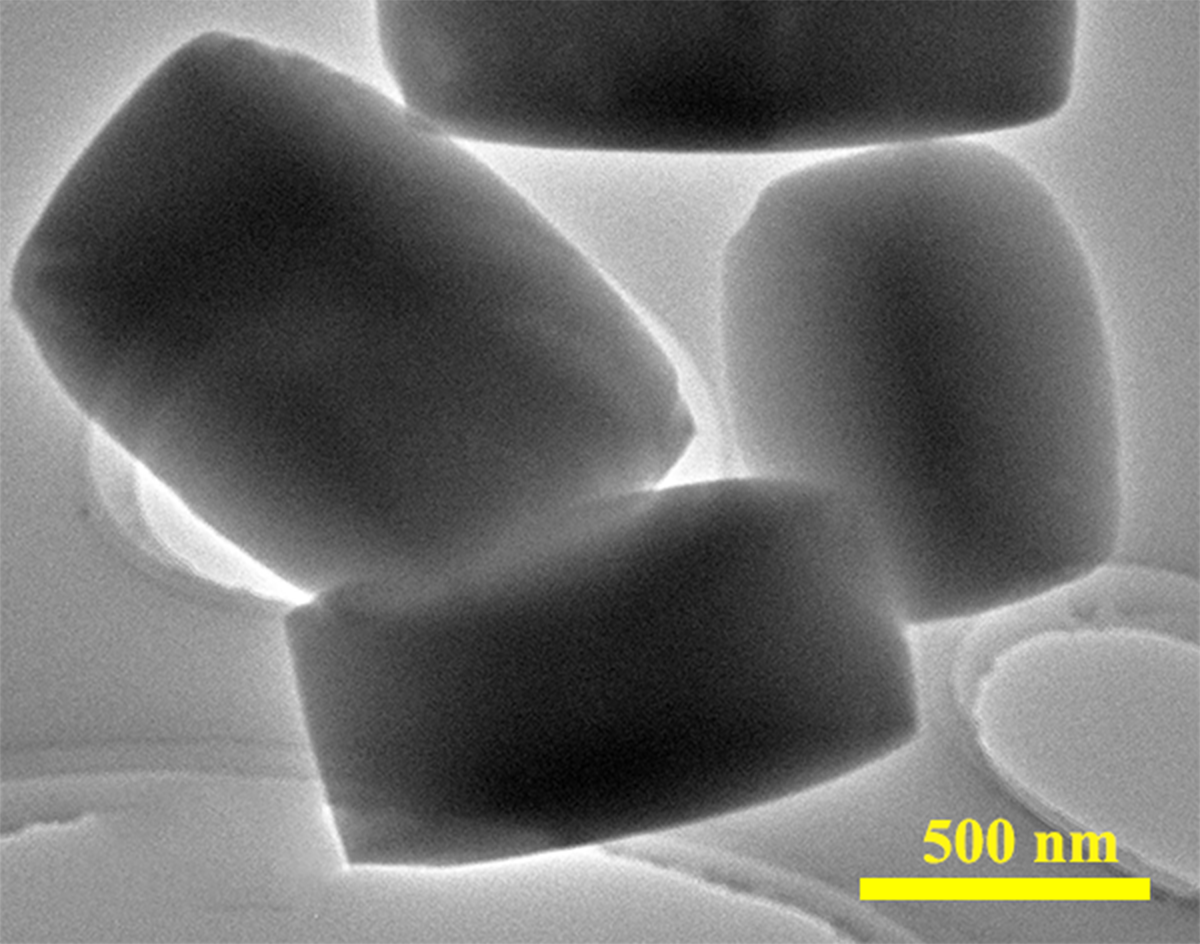


**Fig. S2** TEM image of NH_2_-Ti-MOF


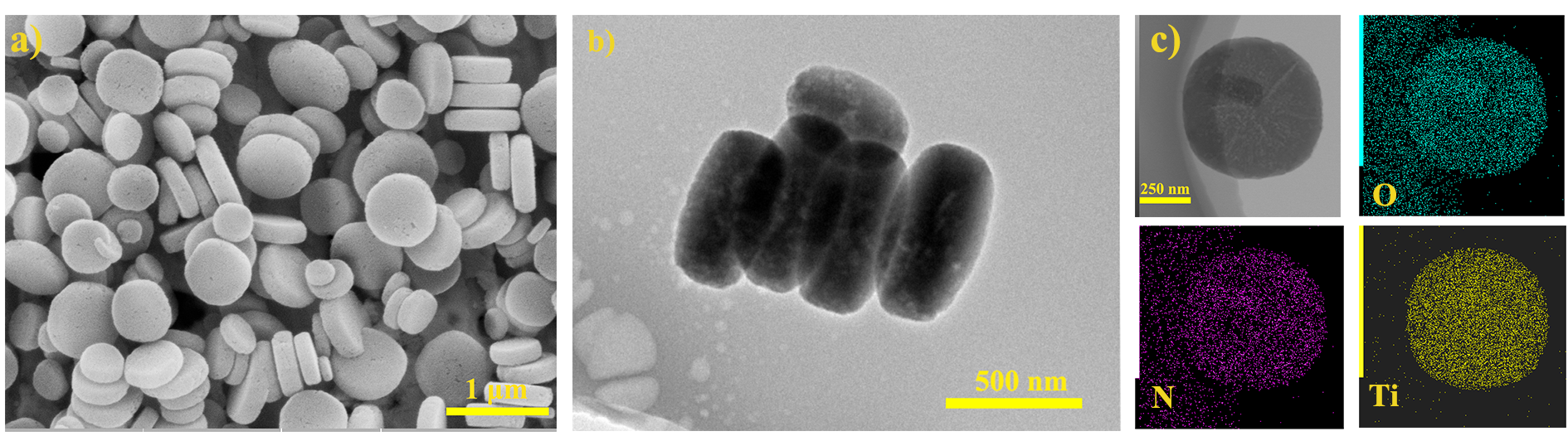


**Fig. S3** TEM image of Ti-MOF


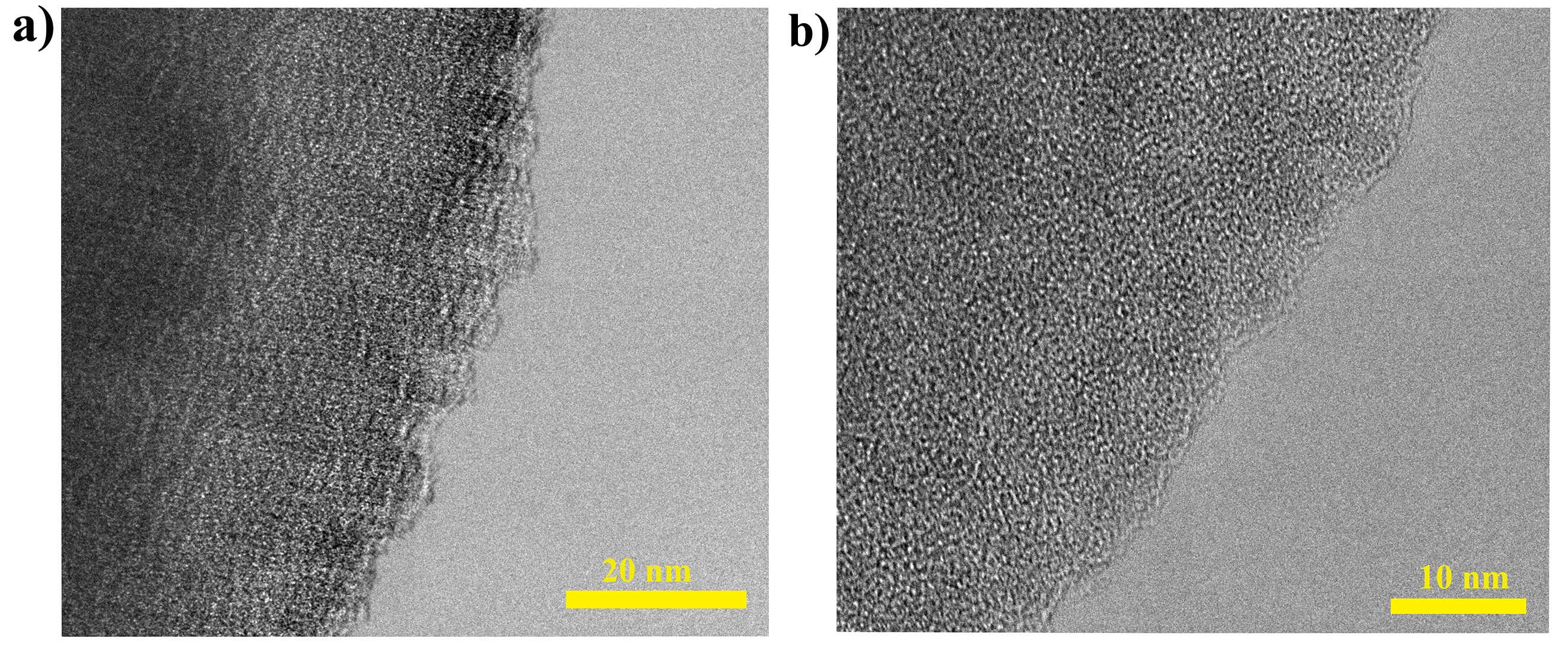


**Fig. S4** High-resolution TEM images clarify the nanoscale porous structure of **a**) NH_2_-Ti-MOF, and **b**) Ti-MOF


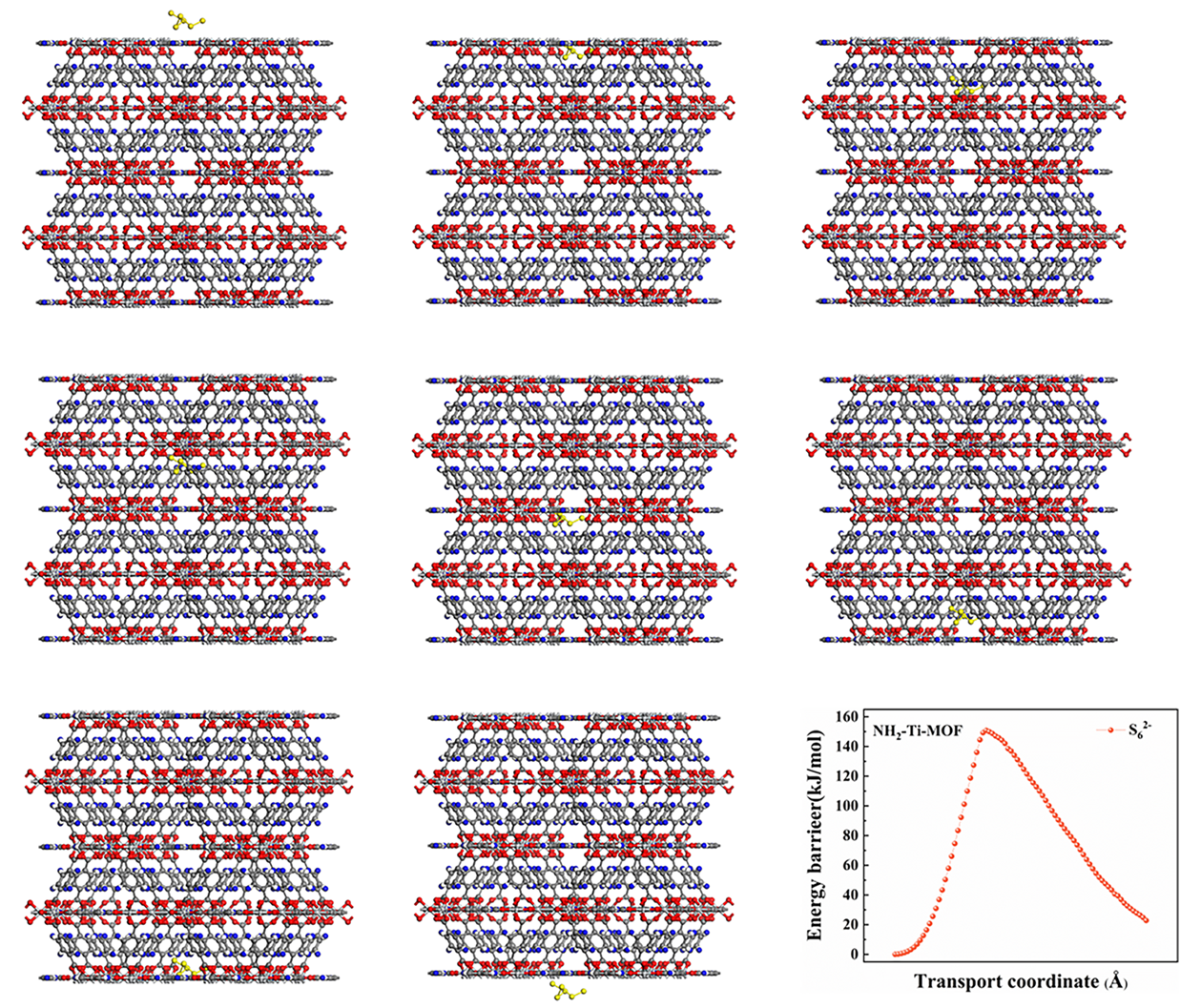


**Fig. S5** Molecular snapshots of S_6_^2-^ at different regions and the energy barrier profiles of S_6_^2-^ during transported NH_2_-Ti-MOF pores


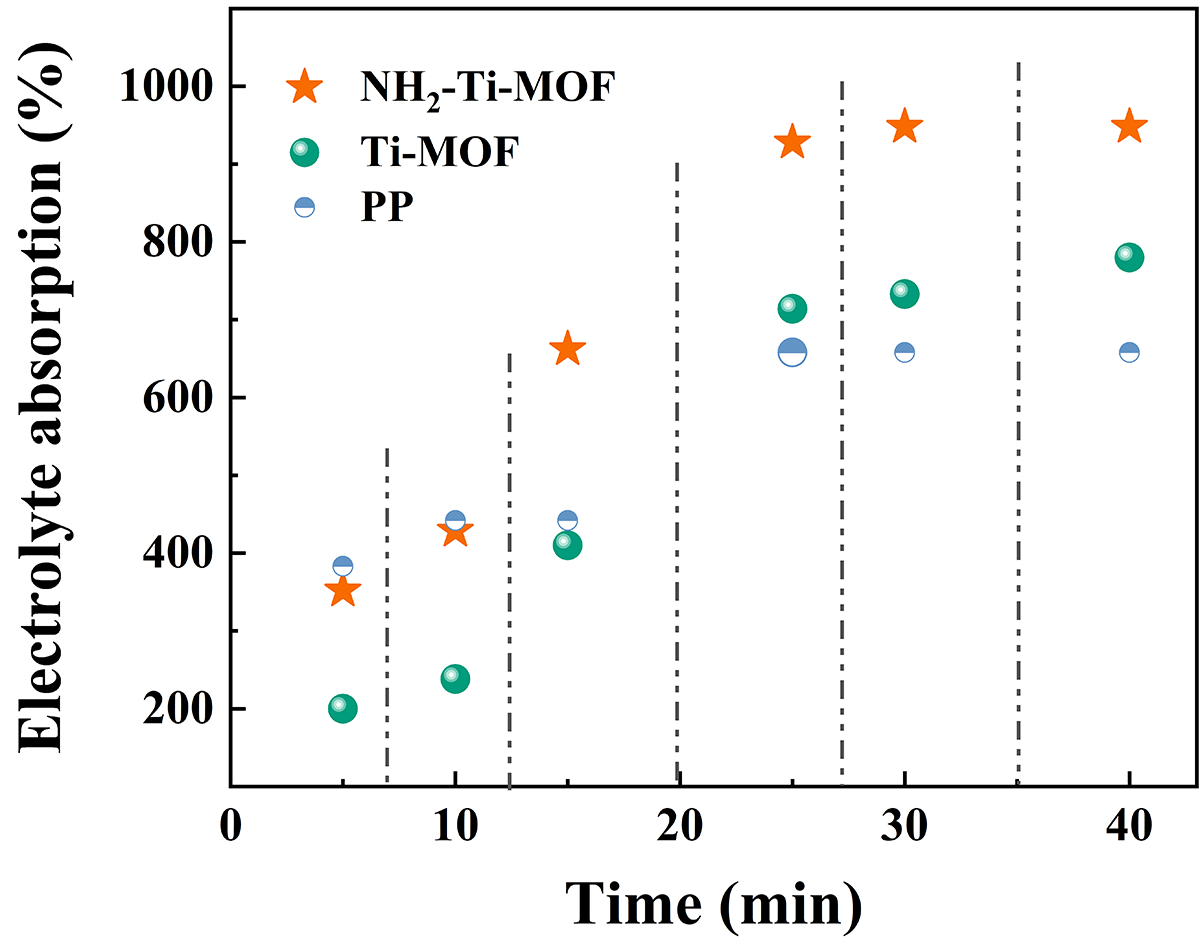


**Fig. S6** Electrolyte uptake of NH_2_-Ti-MOF, Ti-MOF modified separator, and PP separator


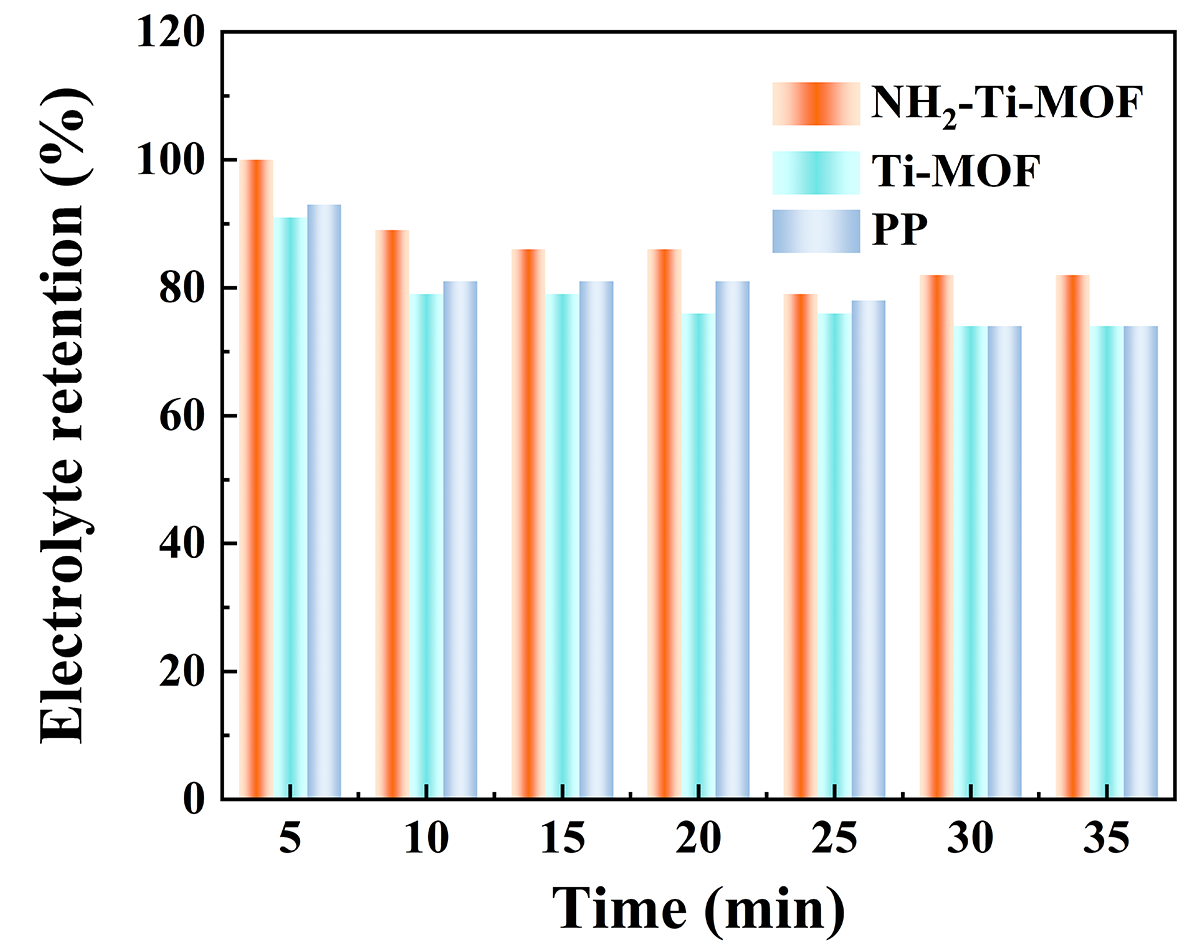


**Fig. S7** Electrolyte retention of NH_2_-Ti-MOF, Ti-MOF modified separator, and PP separator


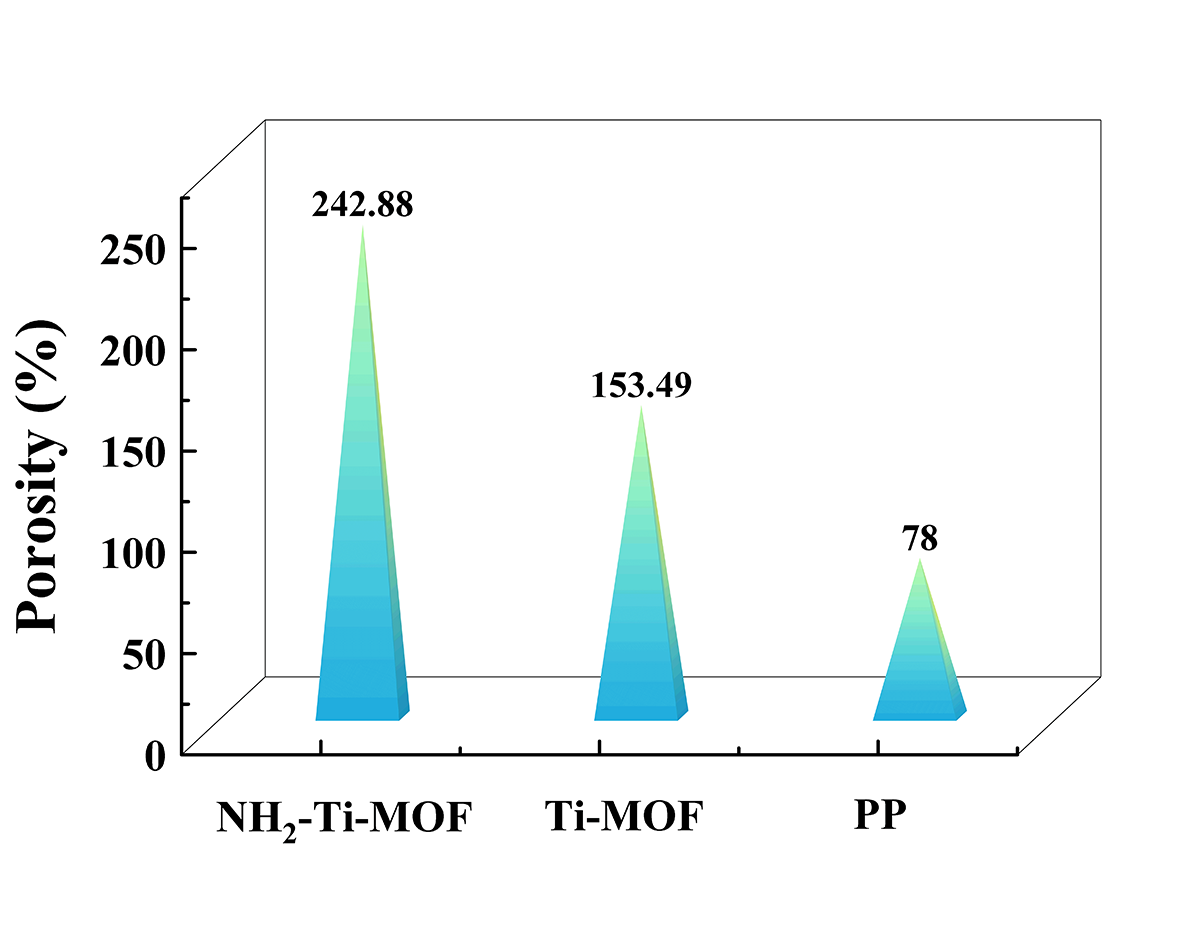


**Fig. S8** Porosity of NH_2_-Ti-MOF, Ti-MOF modified separator, and PP separator

***
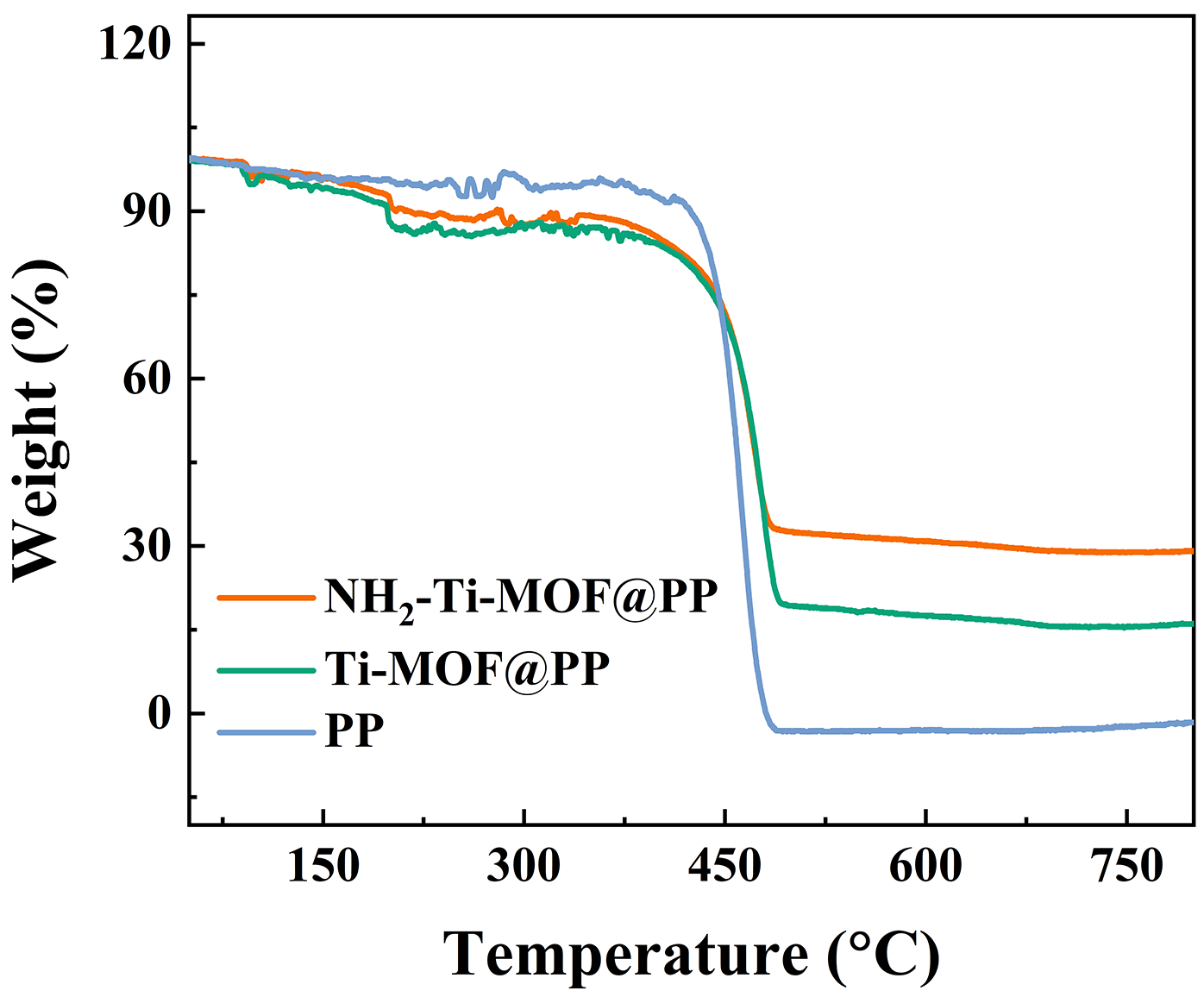
***

**Fig. S9** TGA curves of NH_2_-Ti-MOF, Ti-MOF modified separator, and PP separator


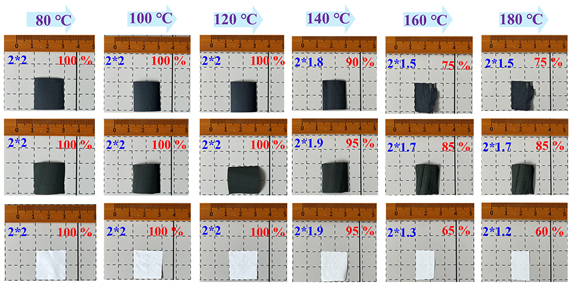


**Fig. S10** Digital photographs of thermal stability tests with pristine PP separator and Ti-MOF, and NH_2_-Ti-MOF modified separators under various temperature settings


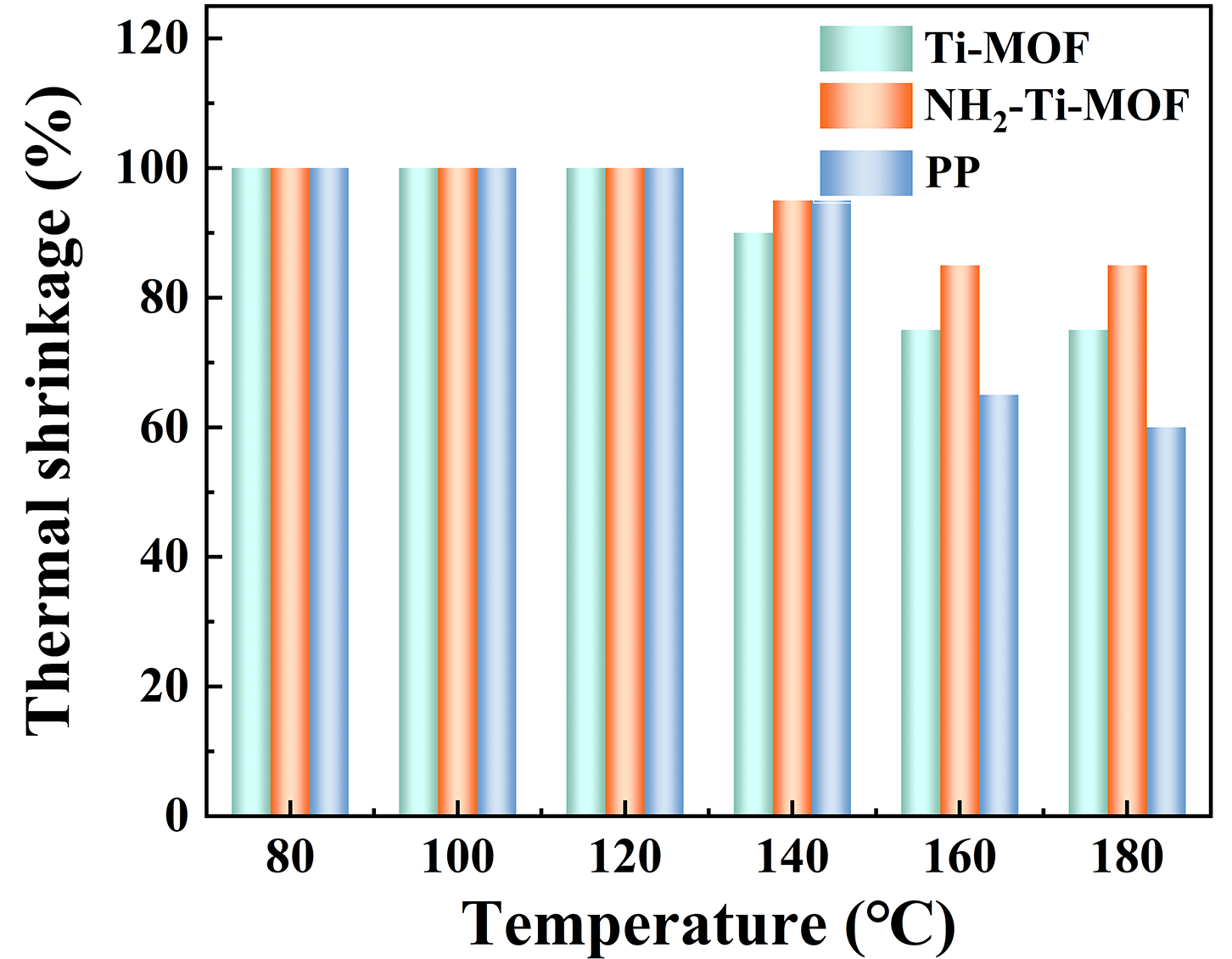


**Fig. S11** Thermal shrinkage with pristine PP separator and Ti-MOF, and NH_2_-Ti-MOF modified separators under different temperatures


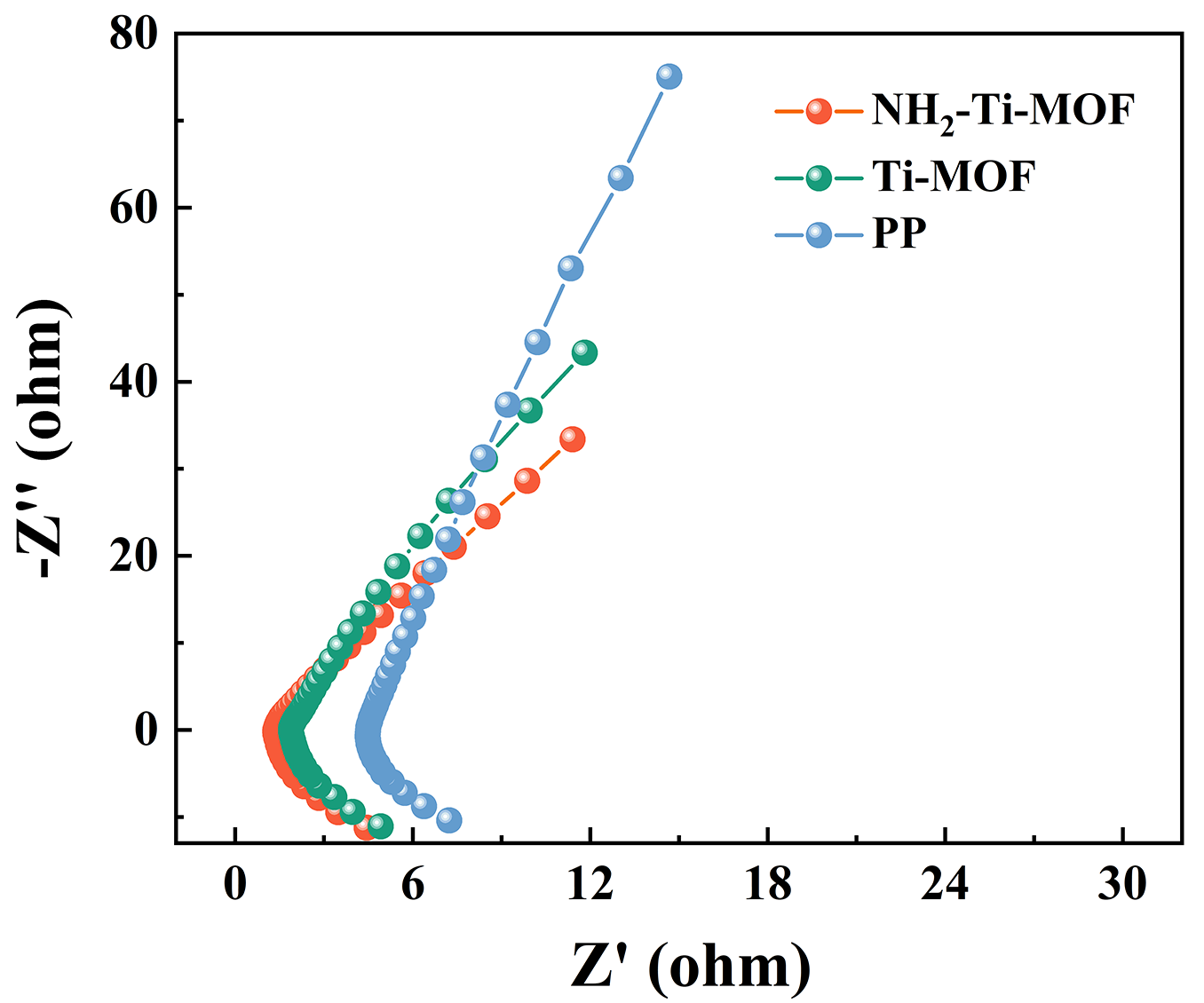


**Fig. S12** The EIS plots of symmetrical batteries with pristine PP separator and Ti-MOF, and NH_2_-Ti-MOF modified separators


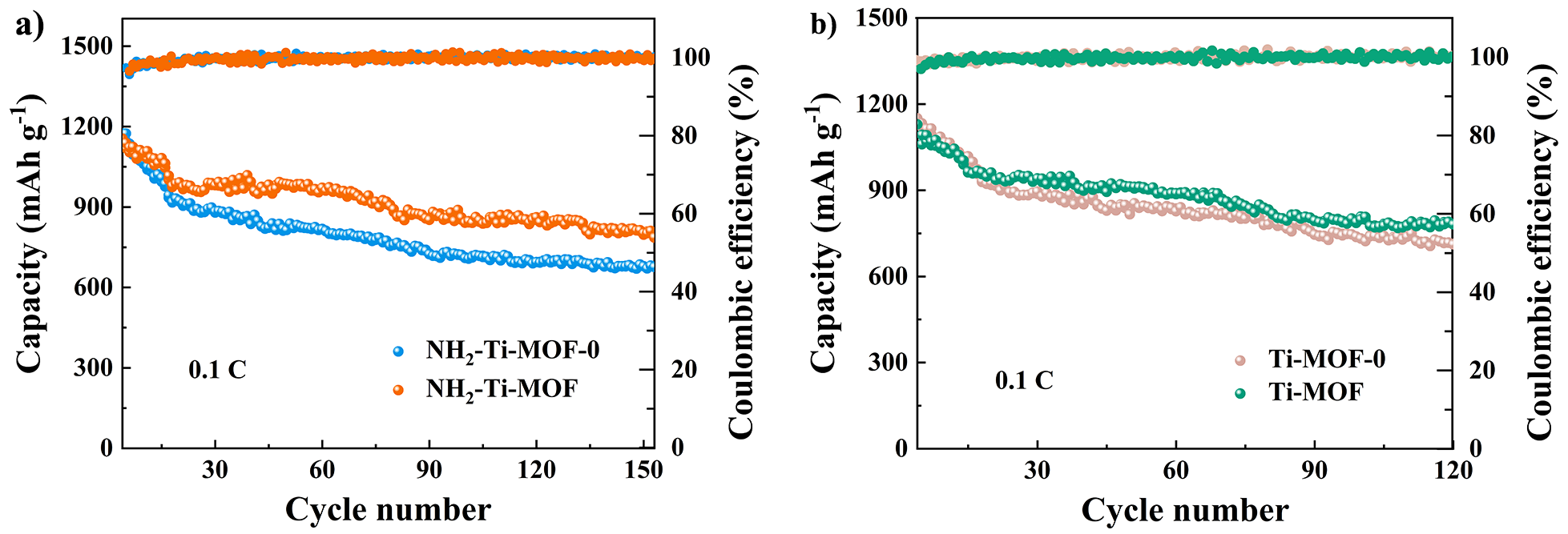


**Fig. S13** Comparison of cycling performances at 0.1 C before and after MOF activation: a) NH_2_-Ti-MOF, and b) Ti-MOF modified separators

**Table S1** The values of capacity fading rate at varied rate

| **Rate (C)** | **0.1 (186 cycles)** | **0.2 (403 cycles)** | **0.5 (603 cycles)** | **1.0 (1003cycles)** | **2.0 (1003 cycles)** |
| --- | --- | --- | --- | --- | --- |
| NH_2_-Ti-MOF | **0.17%** | **0.106%** | **0.07%** | **0.045%** | **0.045%** |
| Ti-MOF | 0.19% | 0.120% | 0.081% | 0.056% | 0.053% |
| PP | 0.48% | 0.200% | 0.13% | 0.073% | 0.059% |

**Table S2** ΔE values with pristine PP separator and Ti-MOF, and NH_2_-Ti-MOF modified separators at different rates

| **Rate (C)** | **0.1** | **0.2** | **0.5** | **1.0** | **2.0** |
| --- | --- | --- | --- | --- | --- |
| △E _(NH2-Ti-MOF)_ | 0.1518 | 0.1534 | 0.2017 | 0.2224 | 0.3054 |
| △E _(Ti-MOF)_ | 0.1725 | 0.1599 | 0.1895 | 0.2514 | 0.3540 |
| △E _(PP)_ | 0.1557 | 0.1533 | 0.1819 | 0.2460 | 0.4670 |


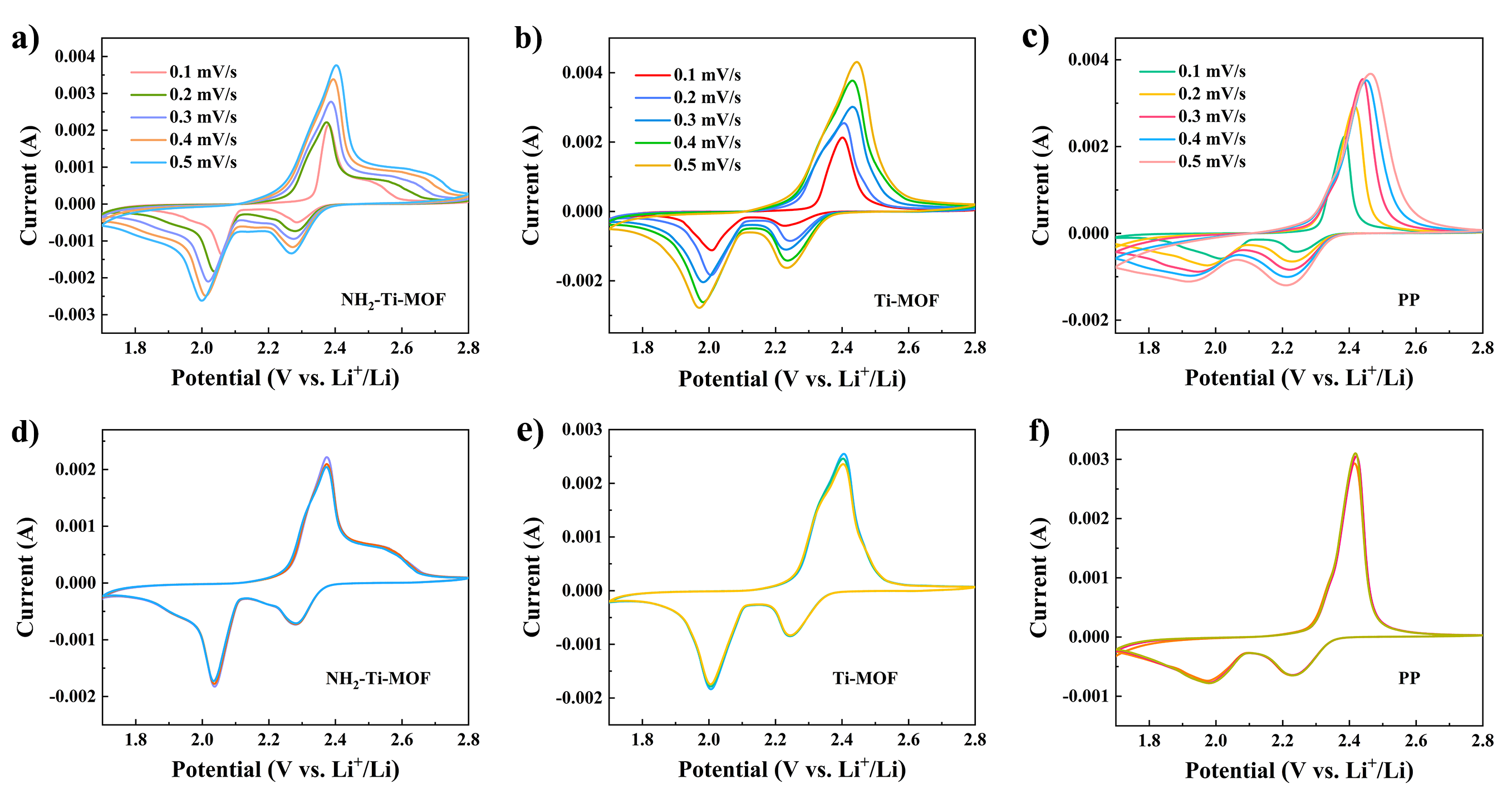


**Fig. S14** CV curves of Li-S batteries at various scanning rates of **a**) NH_2_-Ti-MOF, **b**) Ti-MOF coated separators, and **c**) bare PP separator, CV curves at 0.2 mV s^-1^ of **d**) NH_2_-Ti-MOF, **e**) Ti-MOF coated separators, and **f**) pure PP


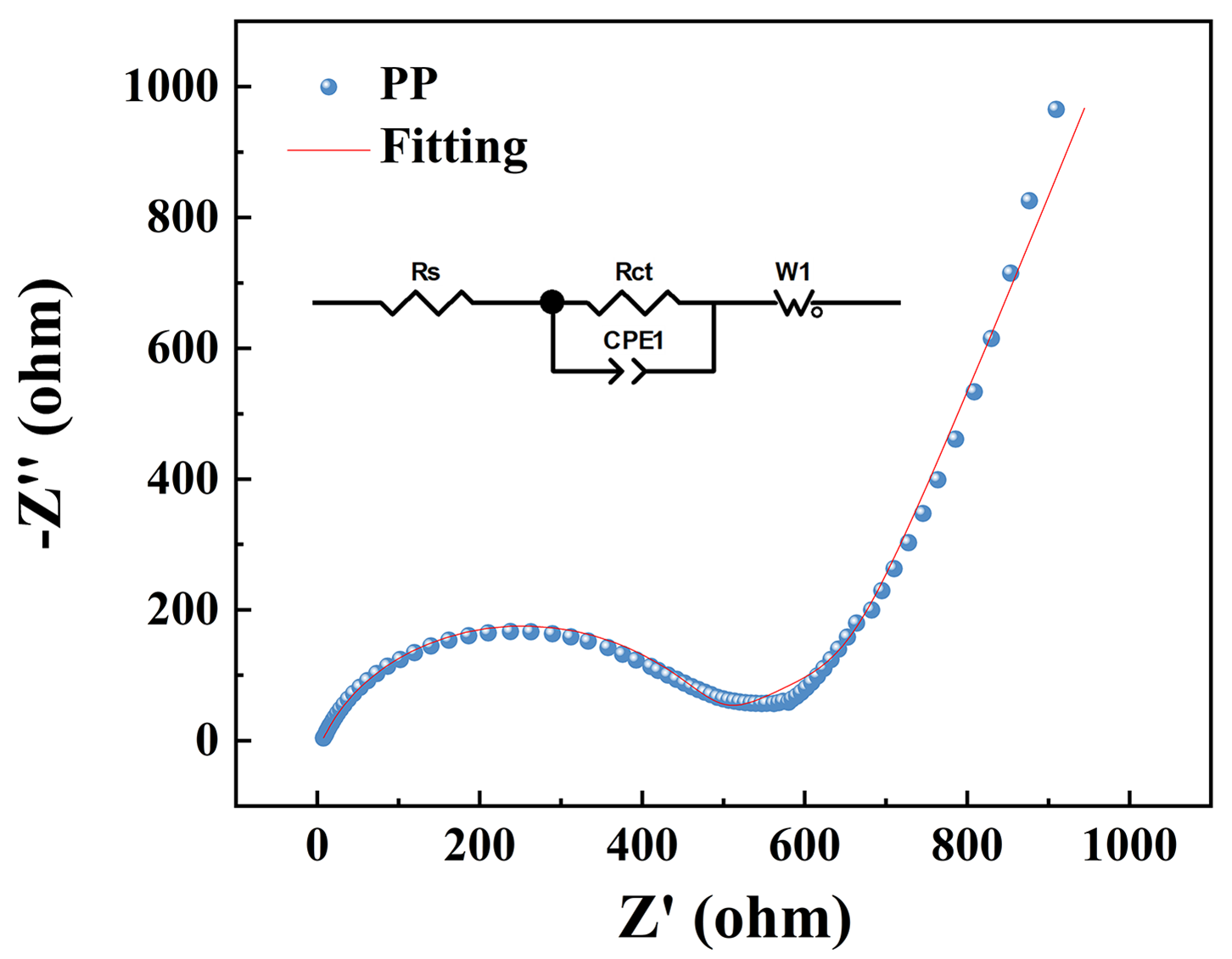


**Fig. S15** EIS plots of Li-S batteries with pristine PP


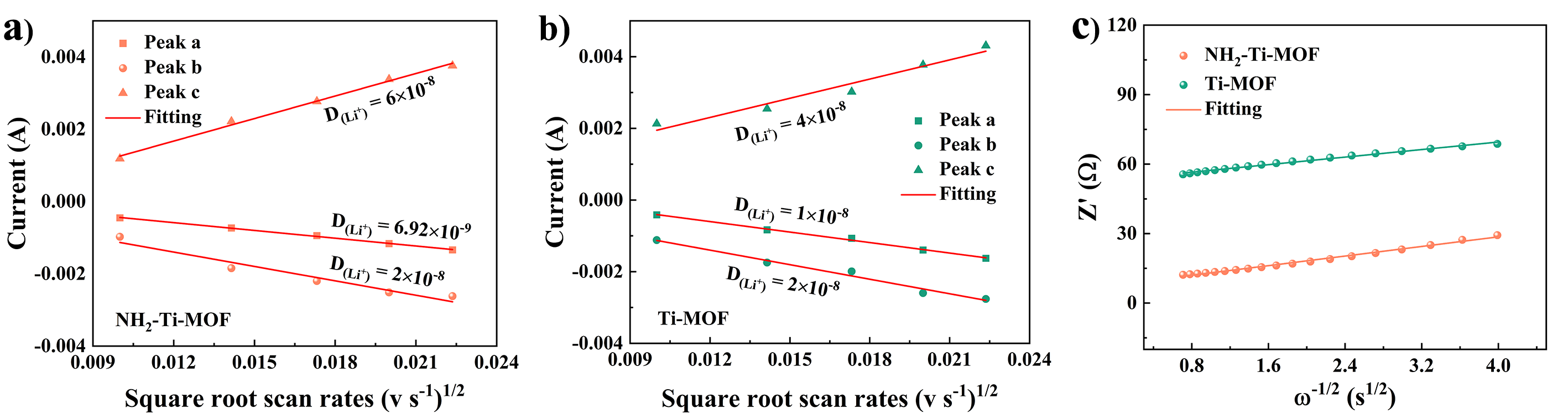


**Fig. S16** Profiles of CV peak currents versus the square root of scan rates for **a**) NH_2_-Ti-MOF and **b**) Ti-MOF, **c**) the relation curves between Z' and ω^-1/2^ of NH_2_-Ti-MOF and Ti-MOF


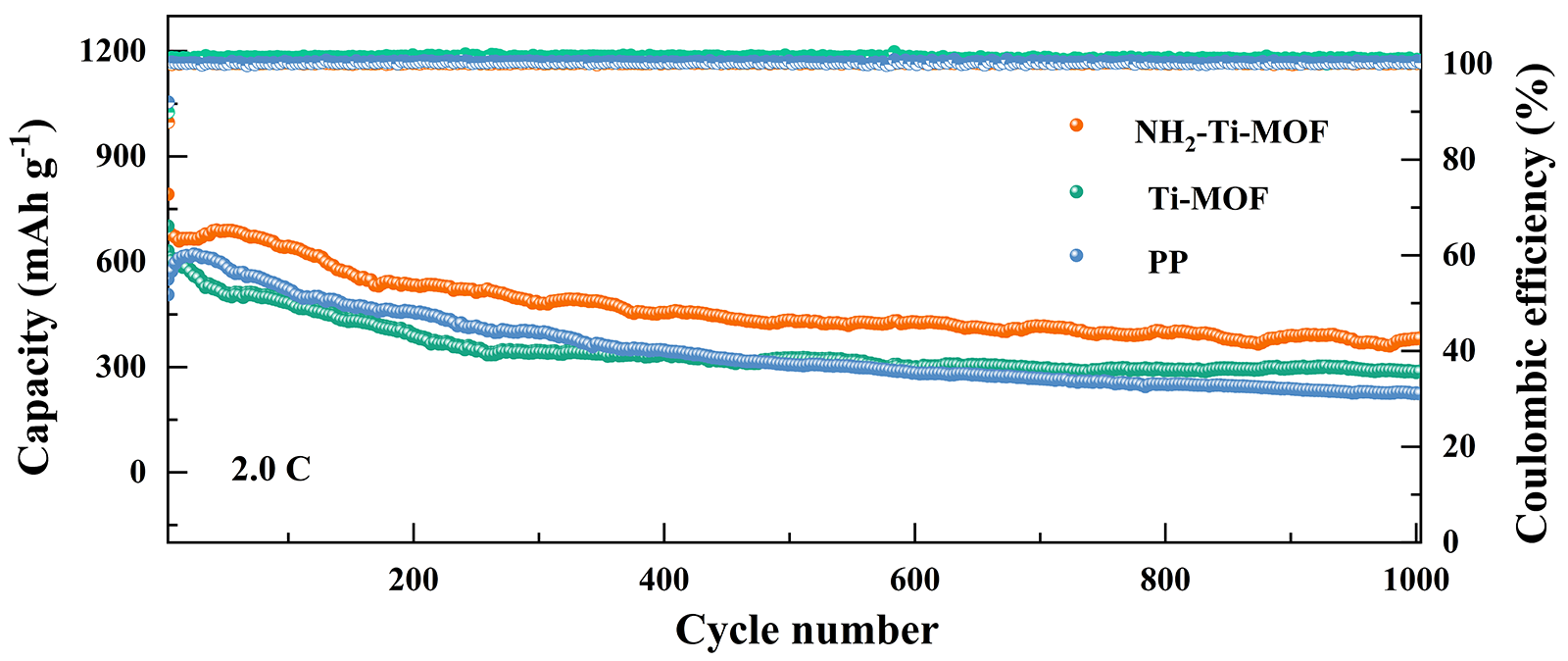


**Fig. S17** Cycle performance of Li-S batteries with different separators at 2.0 C


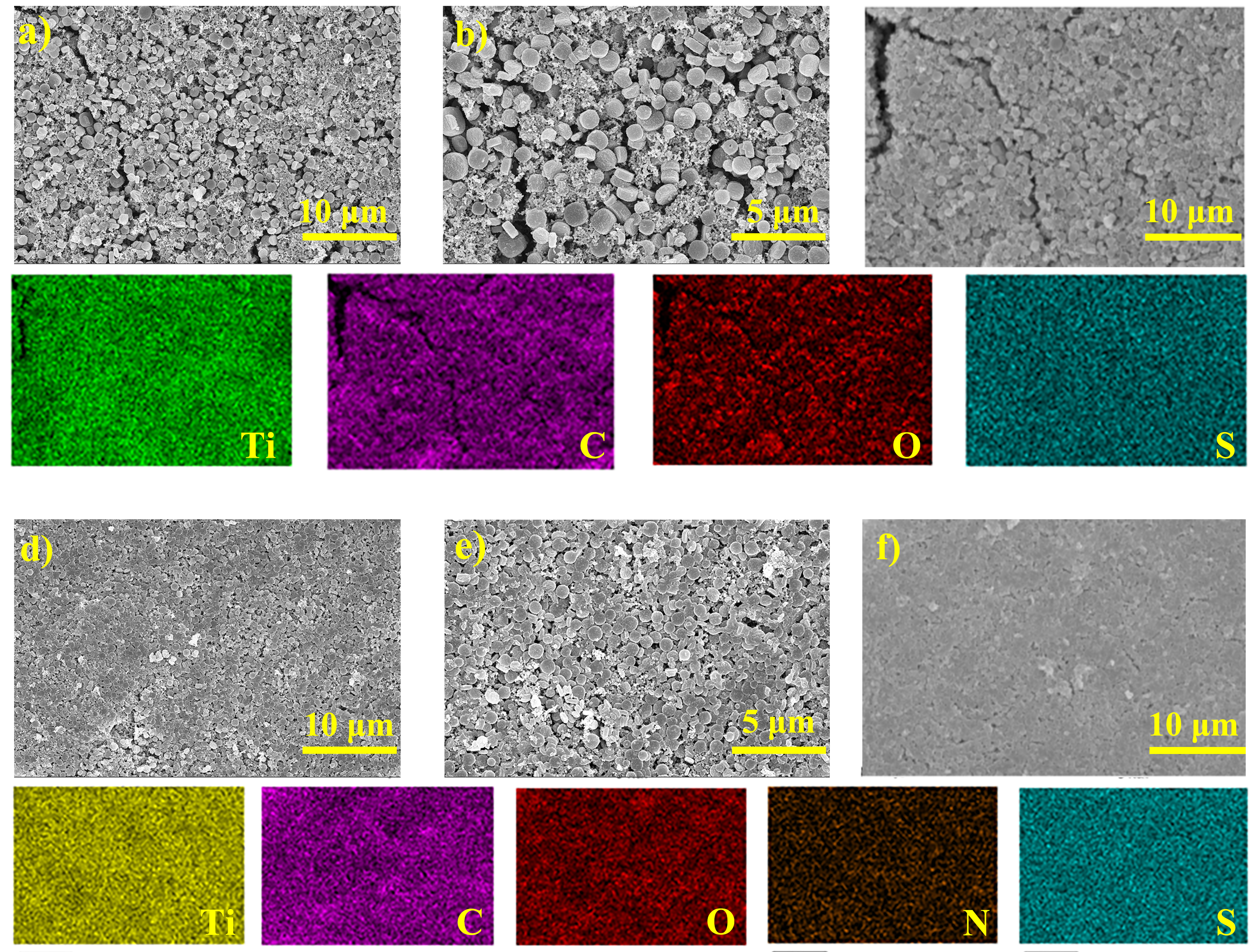


**Fig. S18** SEM images and EDS mapping of **a-c** Ti-MOF and **d-f** NH_2_-Ti-MOF after cycling


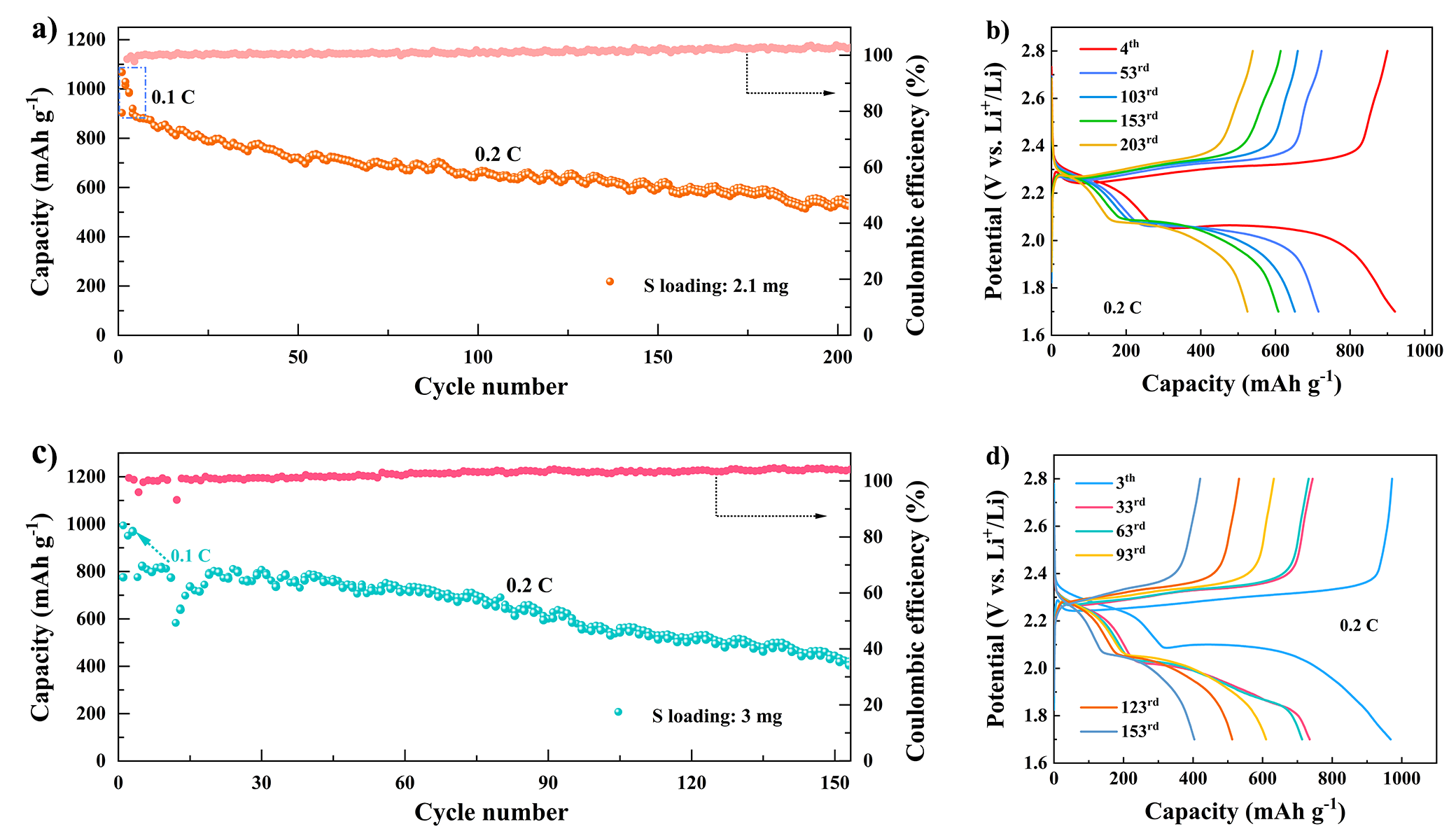


**Fig.** **S19** Electrochemical performances of Li-S batteries with different high loading S as cathode using NH_2_-Ti-MOF coated separator: **a** and **c**) cyclic capability, **b** and **d**) charging/discharging curves at 0.2 C


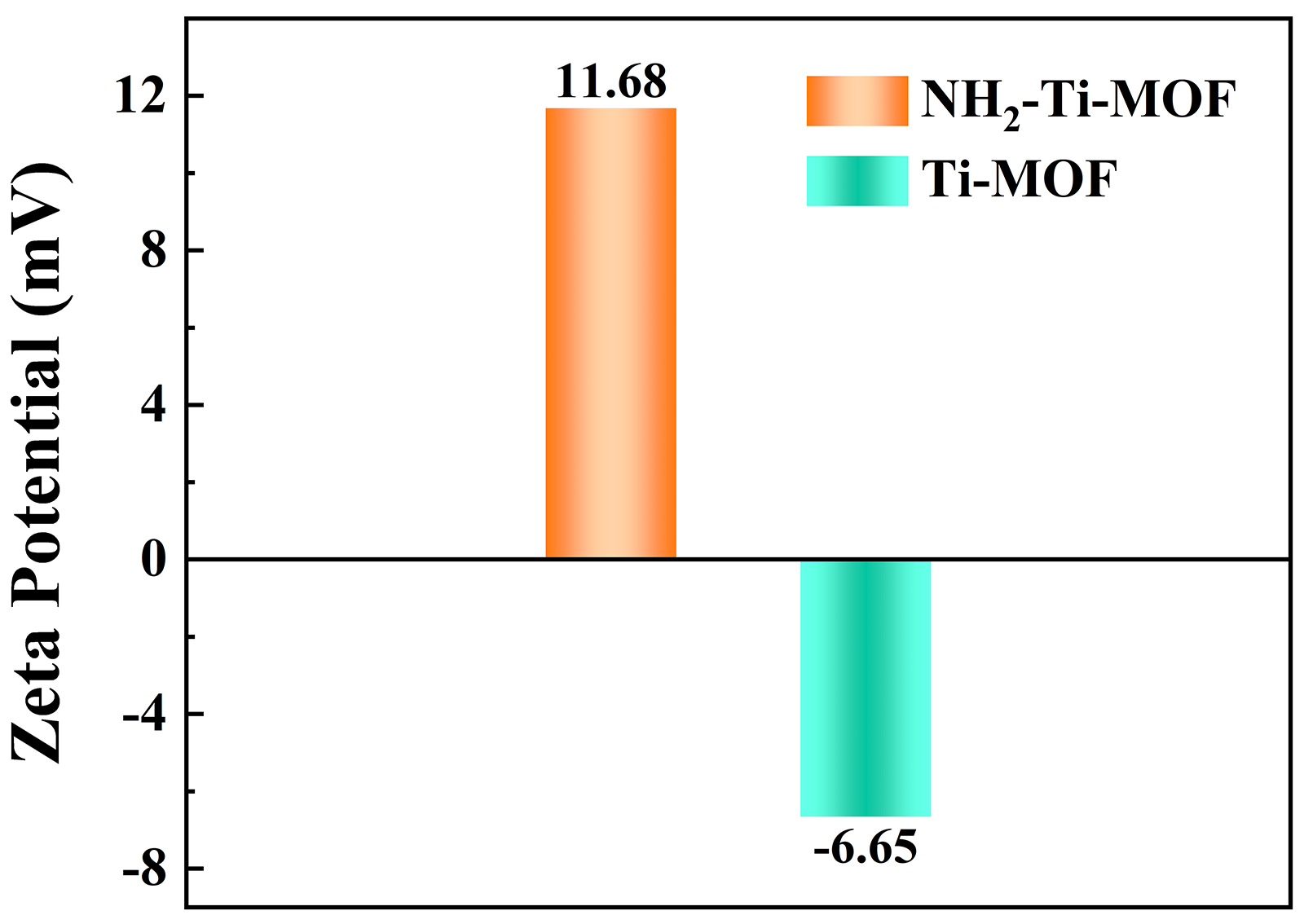


**Fig. S20** Zeta potential of NH_2_-Ti-MOF and Ti-MOF in the electrolyte of Li-S batteries
